# Design of a series of cocrystals featuring isoniazid modified with diacetone alcohol

**DOI:** 10.1107/S2052520622009532

**Published:** 2022-11-08

**Authors:** Matthew Clarke Scheepers, Andreas Lemmerer

**Affiliations:** a Molecular Sciences Institute, School of Chemistry, University of the Witwatersrand, Private Bag 3, Johannesburg, Gauteng 2050, South Africa; CSIR–National Chemical Laboratory, India

**Keywords:** isoniazid, cocrystal, covalent-assisted supramolecular synthesis, crystal structure, pharmaceutical

## Abstract

Eight cocrystals containing an isoniazid derivative derivatized with diacetone alcohol [*N*′-[(2*E*)-4-hy­droxy-4-methyl­pentan-2-yl­idene]pyridine-4-carbohydrazide, **iz4h4m2p**] were synthesized and characterized. All coformers used in this work feature at least one carb­oxy­lic acid functional group. These coformers include: succinic acid, 3,5-di­nitro­benzoic acid, 4-nitro­benzoic acid, 2-chloro-4-nitro­benzoic acid, 2,5-di­hydroxy­benzoic acid, 3-hy­droxy­benzoic acid, 4-hy­droxy­benzoic acid and cinammic acid. Crystal structures were obtained using single crystal X-ray diffraction, which shows that cocrystals containing **iz4h4m2p** feature a similar chain hydrogen bond between molecules of **iz4h4m2p** in addition to the expected carb­oxy­lic acid⋯pyridine hydrogen-bond interaction. Differences between the structures arise when additional hydroxyl group(s) are present, such as the case featuring the hy­droxybenzoic acids. These structures are compared to their isoniazid counterparts presented previously in the literature. All cocrystals were also characterized with differential scanning calorimetry and Fourier transform infrared spectroscopy.

## Abstract 2

Eight cocrystals featuring an isoniazid derivative are synthesized and characterized. Most of the cocrystals show great similarity in hydrogen bonding between each other with only a few exceptions.

## 1. Introduction

Isoniazid or isonicotinic acid hydrazide (**inh**) is an antibacterial prodrug, often used together with a combination of other drugs as part of an effective treatment for tuberculosis (TB). Originally it was combined with *p*-amino­salicylic acid and streptomycin (Iseman, 2002 ▸). **inh** itself is a fairly simple prodrug molecule consisting of primarily a pyridine ring and a hydrazine group. It is converted into the active isonicotinic acyl radical form by the catalase–peroxidase enzyme KatG present in *Mycobacterium tuberculosis* (de Faria *et al.*, 2021 ▸). When treating TB, a single oral dose which contains all the necessary active pharmaceutical ingredients (APIs) in a fixed-dose combination (FDC) is preferred. However, it is known that **inh** can degrade in the presence of other APIs (Bhutani *et al.*, 2004 ▸; Diniz *et al.*, 2018 ▸). As such, this proves to be problematic. Several different strategies can be used to solve this problem.

One of these potential solutions is to use cocrystals. Although no universal agreement of the exact definition of a cocrystal exists, several proposed definitions are available. The definition of a cocrystal proposed by the FDA is: ‘*Solids that are crystalline materials composed of two or more molecules in the same crystal lattice.*’ (Aitipamula *et al.*, 2012 ▸). Aitipamula *et al.* (2012 ▸) proposed several different definitions that included the presence of ions to avoid separating cocrystals and salts from falling under different regulations. Grothe *et al.* (2016 ▸) proposed that a cocrystal is a ‘*crystal with a coformer molecule plus either another coformer or at least two ions*’ with further classifications into different classes. Cocrystals offer the advantage in that the intermolecular interactions between API and coformer can be planned, in addition to the opportunity to patent new intellectual properties (Oruganti *et al.*, 2016 ▸). Cocrystals can potentially offer the advantage of improved properties such as solubility, bioavailability and dissolution rates over the pure material (Blagden *et al.*, 2007 ▸; Ganie *et al.*, 2022 ▸). For pharmaceutical cocrystals the ideal strategy is to use a Generally Regarded As Safe (GRAS) or a pharmaceutically-accepted compound which can form complementary hydrogen bonds with the targeted API (Trask, 2007 ▸). However, in practise, this strategy may prove difficult, as the number of suitable GRAS or pharmaceutically acceptable compounds that could be used is limited. Even if a potentially suitable conformer is found, cocrystallization may not occur due to unknown factors such as kinetic and/or thermodynamic barriers (Bučar *et al.*, 2013 ▸). Therefore, it becomes imperative to explore the structural landscape of new and already existing APIs.

One approach to finding a product with improved properties is to modify an already existing API. Although modifying an API would involve starting clinical trials from scratch, it may prove worthwhile in the long term if the modified API is better than the original form. From a crystal engineering perspective, the modification is a crystal engineering opportunity that can either simplify complex intermolecular interactions or introduce functional groups that can become involved in new intermolecular interactions. For **inh** the hydrazine group is targeted because it can be easily modified (Hearn & Cynamon, 2004 ▸). One of these modifications involves a Schiff-base condensation reaction between **inh** and a ketone or aldehyde of choice. In particular, derivatives using 2-propanone (Wang *et al.*, 2008 ▸; Lemmerer, 2012 ▸; Oruganti *et al.*, 2016 ▸; Boles *et al.*, 2021 ▸) or 2-butanone (Lemmerer *et al.*, 2011 ▸; Madeley *et al.*, 2019 ▸; Setshedi & Smith, 2021 ▸) as the ketones and aldehydes (Ganie *et al.*, 2022 ▸) including salinazid (Surov *et al.*, 2016 ▸) have been reported in the literature in crystal engineering studies. This changes the hydrazide group to an amide and an imine group. From a crystal engineering perspective, these modified **inh** can have a reduced number of hydrogen bond donors. Although this may reduce the chances of forming a cocrystal since the only hydrogen pair donor remaining will be the amide and hydrogen bond acceptors will be the oxygen from the amide and the pyridine ring; however, this should not be a big problem, as carb­oxy­lic acids have been shown to form a robust interaction with pyridines (either a hydrogen bond or a proton transfer) very easily (Shattock *et al.*, 2008 ▸).

**Figure 1:**
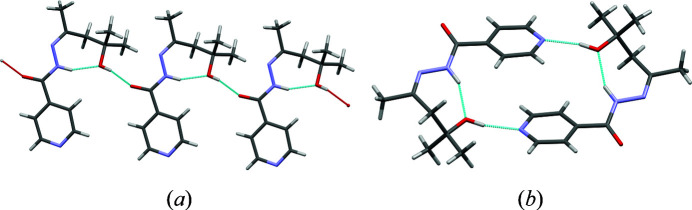
The structures of **iz4h4m2p** and the different hydrogen-bonding schemes between (*a*) form **I** and (*b*) form **II**.

In a previous work (Scheepers *et al.*, 2022 ▸), we presented *N*′-[(2*E*)-4-hy­droxy-4-methyl­pentan-2-yl­idene]pyridine-4-carbohydrazide (**iz4h4m2p**), which is **inh** derivatized using diacetone alcohol (4-hy­droxy-4-methyl-2-pentanone) *via* a Schiff-base condensation reaction. This ketone consists of a five carbon length alkyl chain with an extra methyl group and a hydroxyl group. This compound was determined to be polymorphic in our previous study (Scheepers *et al.*, 2022 ▸), consisting of a metastable form, form **I** and a stable form, form **II** (Scheepers *et al.*, 2022 ▸). The major difference between the two forms is the different hydrogen bonding present in each form: form **I** forms a chain hydrogen-bond motif between the hydroxyl group to the oxygen atom from the amide group from a neighbouring **iz4h4m2p** molecule, while form **II** forms a ring hydrogen-bond motif where the hydroxyl group forms a hydrogen bond to the pyridine ring, which subsequently combines to form dimers (Fig. 1 ▸). Like the other hydrazone derivatives of **inh** mentioned, **iz4h4m2p** still features a bulky alkyl chain backbone. However, the newly introduced hydroxyl group should allow for some variation from the previously observed cocrystals of the hydrazone derivatives of **inh**. Since **iz4h4m2p** contains a pyridine, it should readily interact with molecules containing a carb­oxy­lic acid group. This interaction can potentially reduce the supramolecular assembly from 2D sheets to a 1D ribbon (Lemmerer *et al.*, 2011 ▸). This increases the chances of forming cocrystals with more predictable supramolecular assemblies.

So far, only one cocrystal containing **iz4h4m2p** has been reported (Madeley *et al.*, 2019 ▸) and it contained 4-*tert*-butyl­benzoic acid. This cocrystal consists of a hydrogen bond formed between the pyridine ring of **iz4h4m2p** and the carb­oxy­lic acid group, while a chain hydrogen-bond motif exists between the hydroxyl group of the **iz4h4m2p** molecule to the oxygen of the amide group from a neighbouring **iz4h4m2p** molecule. Based on this, would other **iz4h4m2p** cocrystals with carb­oxy­lic acid coformers form similar structures with similar hydrogen-bond motifs? Will additional hydrogen-bond donors/acceptors from the coformers have a considerable effect on the structure of these cocrystals? Hence, the objective for this work is to explore the multicomponent crystal landscape of **iz4h4m2p**. This involves designing and synthesizing cocrystals of **iz4h4m2p** with coformers containing the carb­oxy­lic acid group.

## 2. Experimental

### 2.1. Materials

All materials were purchased from Sigma-Aldrich and were used as is without further purification.

### 2.2. General procedure for synthesis of **iz4h4m2p** and its cocrystals

**iz4h4m2p** was synthesized using a previously described method. **inh** and diacetone alcohol were dissolved in absolute ethanol (5 ml) in a small glass vial containing a magnetic stirrer bar. This vial was closed and stirred for 24 h at room temperature (∼295–300 K). After which, the lid of the vial was replaced with another lid with a small hole in it to allow solvent to evaporate out. After several days crystals of **iz4h4m2p** were obtained. These crystals were of form **II**.

The general procedure for the synthesizing cocrystals was as follows: stoichiometric amounts of **iz4h4m2p** and the respective coformer were dissolved in an appropriate solvent/solvent combination in a small glass vial at room temperature. The vial is closed using a lid with a small hole in it to allow solvent to evaporate out. Crystals are obtained after several days.

### 2.3. Powder X-ray diffraction

Powder X-ray diffraction (PXRD) is used to determine the bulk phase purity of each sample. PXRD data for all forms were measured at 293 K on a Bruker D2 Phaser diffractometer which employs a sealed tube Co X-ray source (λ = 1.78896 Å), operating at 30 kV and 10 mA, and LynxEye PSD detector in Bragg–Brentano geometry. Powder patterns for the cocrystals are presented in the supporting information, where the experimentally measured pattern is compared to the calculated patterns obtained from the SC-XRD data.

### 2.4. Single crystal X-ray diffraction

A Bruker D8 Venture Photon CMOS 100 area detector diffractometer, equipped with a graphite-monochromated Mo *K*α_1_ (λ = 0.71073 Å) sealed tube (50 kV, 30 mA), was used to collect all the intensity data. Crystal structures were determined at 173 K. The program *SAINT+* (version 6.02) (Bruker, 2012 ▸) was used to integrate the data and the program *SADABS* (Krause *et al.*, 2015 ▸) was used to make empirical absorption corrections. Space group assignments were made using *XPREP* (Bruker, 2012 ▸) for all compounds. In all cases except for the case of **iz4h4m2p** + **4hba**, the structures were solved in the *WinGX* suite of programs (Farrugia, 1999 ▸) by intrinsic phasing using *SHELXT* (Sheldrick, 2015 ▸) and refined using full-matrix least-squares/difference Fourier techniques on *F* ^2^ using *SHELXL* (Sheldrick, 2018 ▸). All non-hydrogen atoms were refined anisotropically. All carbon-bound hydrogen atoms were placed at idealized positions and refined as riding atoms with the *U*_iso_ parameter 1.2 or 1.5 times that of their parent atoms. Nitro­gen-bound and oxygen-bound hydrogen atoms were located in a difference Fourier map and their coordinates and isotropic displacement parameters refined freely. *Olex2* (version 1.3) (Dolomanov *et al.*, 2009 ▸) was used to solve the disordered system of **iz4h4m2p** + **4hba**. Diagrams and publication material were generated using *ORTEP-3* (Farrugia, 2012 ▸) and *MERCURY* (Macrae *et al.*, 2020 ▸). The crystallographic information can be found in Table 1 ▸.

### 2.5. The Cambridge Structural Database

The Cambridge Structural Database (CSD version 2022.1.0) (Groom *et al.*, 2016 ▸) was used to compare the cocrystals presented in this work with the cocrystals of **inh**, as well as the cocrystals of any notable **inh** derivative. The only restriction was that entries must be classified as being organic. *MERCURY* (Macrae *et al.*, 2020 ▸) was used to inspect the crystal structures. The aromatic analyser tool of *MERCURY* was used to inspect the aromatic interactions of the aryl rings. The crystal structure similarity tool was used to compare the structural similarity of selected structures.

### 2.6. Differential scanning calorimetry

Differential scanning calorimetry (DSC) data were collected using a Mettler Toledo DSC 3 with aluminium pans under nitro­gen gas (flow rate = 10 ml min^−1^). Exothermic events were shown as peaks. Samples were heated and cooled to determine melting points as well as any additional phase transitions. The temperature and energy calibrations were performed using pure indium (purity 99.99%, m.p. 156.6°C, heat of fusion: 28.45 J g^−1^) and pure zinc (purity 99.99%, m.p. 479.5°C, heat of fusion: 107.5 J g^−1^). Samples were heated to 250°C from 25°C before being cooled back down at 25°C at a heating or cooling rate of 10°C min^−1^.

### 2.7. Fourier transform infrared analysis

FTIR spectra were collected using the Bruker Alpha II spectrometer equipped with an Eco-ATR sampling module. Background noise was subtracted and a small amount of the sample of interest was placed onto the ATR crystal. Spectra were measured in the 600 to 4000 cm^−1^ range, resolution was 4 cm^−1^ with 24 scans per sample. Spectra were collected in a room with air conditioning set at 295 K. After spectral acquisition the ATR crystal was cleaned using iso­propanol.

## 3. Results and discussion

### 3.1. Obtaining cocrystals

**Figure 2:**
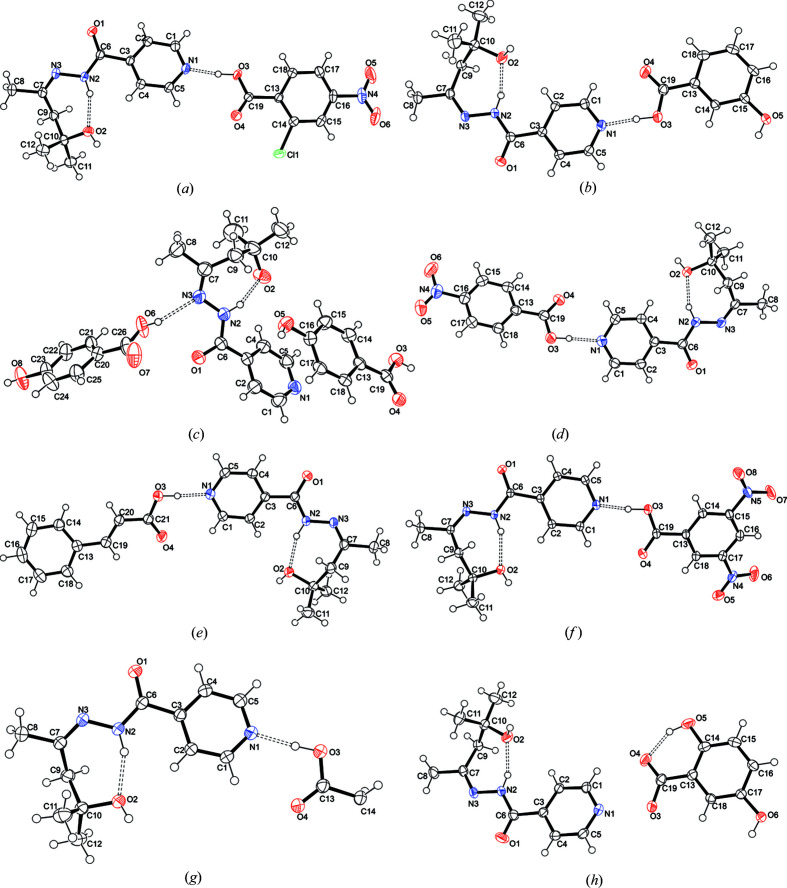
*ORTEP* diagrams of (*a*) **iz4h4m2p** + **2c4n**, (*b*) **iz4h4m2p** + **3hba**, (*c*) **iz4h4m2p** + **4hba**, (*d*) **iz4h4m2p** + **4nba**, (*e*) **iz4h4m2p** + **ca**, (*f*) **iz4h4m2p** + **dnba**, (*g*) **iz4h4m2p** + **sa** and (*h*) **iz4h4m2p** + **2,5-dhba**.

In order to obtain cocrystals containing **iz4h4m2p** with molecules containing at least one carb­oxy­lic acid, several coformers were used. Most of these consist of Generally Regarded As Safe (GRAS) compounds, such as succinic acid (**sa**) and gentisic acid (2,5-di­hydroxy­benzoic acid, **2,5-dhba**). These coformers include ones which have either one or two carb­oxy­lic acid groups. A full list of all the coformers used is included in the supporting information. For this work, we used various stoichiometric ratios such as 1:1, 2:1 and 1:2 of **iz4h4m2p**: coformer with different solvents (methanol, ethanol, aceto­nitrile, water) and solvent mixtures (1:1 ratios of solvent 1:solvent 2). It should be noted that we avoided heating the solution, as **iz4h4m2p** can decompose in a hot solution, resulting in an oil. Thus we relied on stirring the solution for 10–20 min at room temperature to ensure complete dissolution. Out of a list of 28 coformers used, only eight formed cocrystals. The reasons why we did not obtain more were due to the possibility that some of the acids still produced oils as opposed to crystals, while in other cases the coformers precipitated out separately without forming a cocrystal. The eight cocrystals that were synthesized used the following coformers: 3,5-di­nitro­benzoic acid (**dnba**), succinic acid, **2,5-dhba**, 3-hy­droxy­benzoic acid (**3hba**), 4-hy­droxy­benzoic acid (**4hba**), 4-nitro­benzoic acid (**4nba**), 2-chloro-4-nitro­benzoic acid (**2c4n**) and cinammic acid. The molar ratios and solvents used to synthesize these are presented in Table 1 ▸. The results are presented below in four separate sections because of some similarities and major differences between certain crystal structures and/or coformers. These sections are: (i) **iz4h4m2p** + **sa** and **iz4h4m2p** + **ca**, (ii) **iz4h4m2p** + **dnba**, **iz4h4m2p** + **2c4n** and **iz4h4m2p** + **4nba**, (iii) **iz4h4m2p** + **4hba**, (iv) **iz4h4m2p** + **2,5-dhba** and **iz4h4m2p** + **3hba**. The reasoning for splitting these cocrystals into these sections is as follows: (i) it was suspected that **iz4h4m2p** + **ca** and **iz4h4m2p** + **sa** to have a similar hydrogen-bonding patterns due to the absence of additional hydrogen bond donors/acceptors, (ii)) in **iz4h4m2p** + **dnba**, **iz4h4m2p** + **2c4n**, and **iz4h4m2p** + **4nba** the coformers all feature a nitro group, which may or may not have had a significant impact on the crystal structure formation while (iii) and (iv) the additional hydrogen bond donors had a significant impact in the overall hydrogen bond interactions observed that made the structures unique in comparison to the structures described in (i) and (ii). The crystal structure information is presented in Table 2 ▸, while *ORTEP* diagrams are presented in Fig. 2 ▸. Graph sets have been used to describe some of the hydrogen bonding observed in the crystal structures (Etter *et al.*, 1990 ▸).

### 3.2. Crystal structures of **iz4h4m2p** + **sa** and **iz4h4m2p** + **ca**

**Figure 3:**
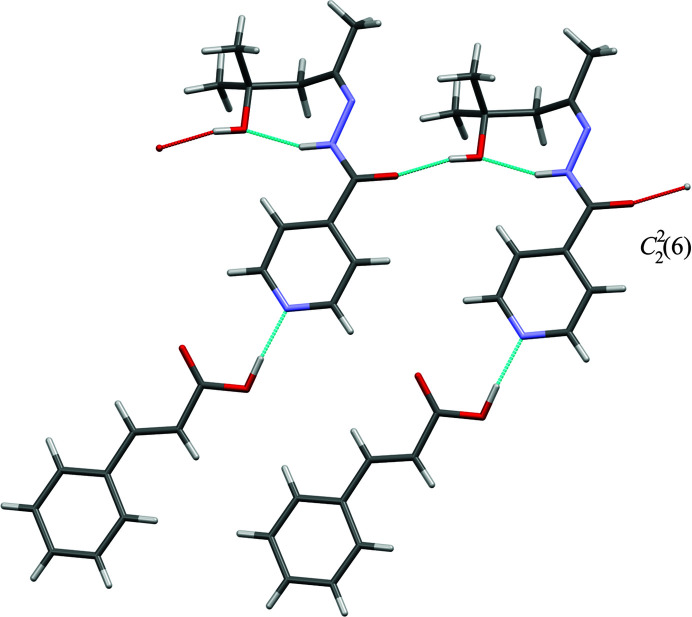
Hydrogen bonding in **iz4h4m2p** + **ca** which is the standard hydrogen-bonding scheme in most of the structures presented.

**Figure 4:**
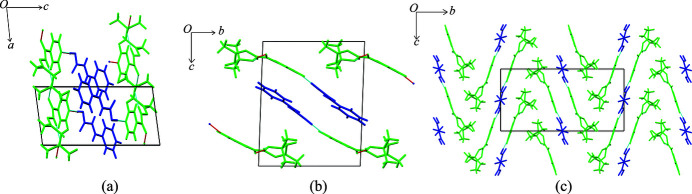
Crystal structure packing of (*a*) **iz4h4m2p** + **ca** (down the *b*-axis), (*b*) **iz4h4m2p** + **ca** (down the *a*-axis) and (*c*) **iz4h4m2p** + **sa** (down the *a*-axis). **Iz4h4m2p** molecules are presented in green while the respective coformers are in blue.

**iz4h4m2p** formed cocrystals with succinic acid and cinammic acid, forming colourless needles and colourless plates, respectively. The asymmetric unit of **iz4h4m2p** + **ca** consists of one molecule each of **iz4h4m2p** and **ca** and crystallizes in space group 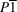 , while the asymmetric unit of **iz4h4m2p** + **sa** consists of one molecule of **iz4h4m2p** and half a molecule of succinic acid (which sits on a special crystallographic site), and crystallizes in space group *P*2_1_/*c*. In each case the respective carb­oxy­lic acid forms a hydrogen bond with the pyridyl nitro­gen of **iz4h4m2p**. Furthermore, this results in the hydroxyl group of **iz4h4m2p** forming a hydrogen bond with the oxygen of the amide from a neighbouring **iz4h4m2p** molecule, which forms a 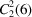 chain hydrogen-bond motif similar to the one observed in form **I** of **iz4h4m2p**. Additionally, for **iz4h4m2p** + **ca**, π⋯π stacking exists between the rings of adjacent **ca** molecules and pyridine rings of **iz4h4m2p** (*Cg*⋯*Cg* = 4.01 Å). Although both crystal structures form 1D ribbons (Fig. 3 ▸), the packing between the two are different. The ribbons in **iz4h4m2p** + **ca** feature a more stacked or layered structure, with cinammic acid and **iz4h4m2p** separated by alternating layers [Figs. 4 ▸(*a*) and 4 ▸(*b*)], while the ribbons from the cocrystal of **iz4h4m2p** + **sa** form a zigzag pattern [Fig. 4 ▸(*c*)].

### 3.3. Crystal structures of **iz4h4m2p** + **dnba**, **iz4h4m2p** + **2c4n** and **iz4h4m2p** + **4nba**

**Figure 5:**
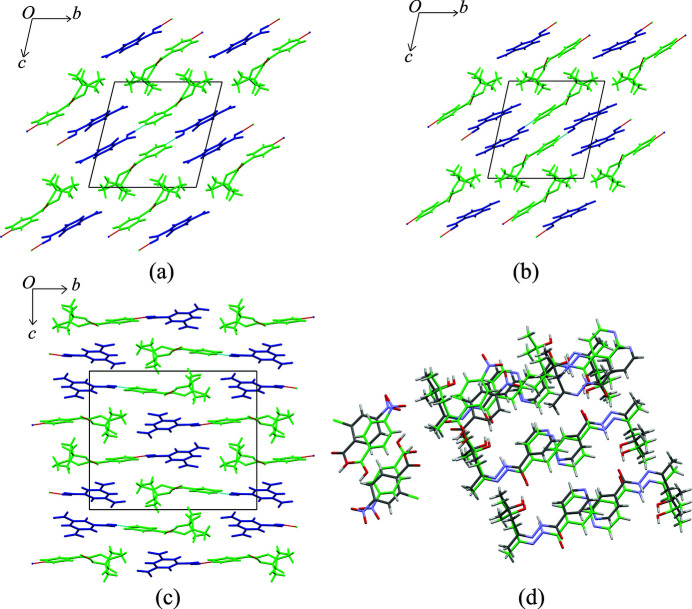
Crystal structure packing of (*a*) **iz4h4m2p** + **2c4n** (viewed down the *a*-axis), (*b*) **iz4h4m2p** + **4nba** (viewed down the *a*-axis), (*c*) **iz4h4m2p** + **dnba** (viewed down the *a*-axis). In (*a*)–(*c*) **iz4h4m2p** molecules are presented in green while the respective coformer is shown in blue. (*d*) The overlay of molecules from **iz4h4m2p** + **4nba** (represented as green molecules) and **iz4h4m2p** + **2c4n** (using standard colours).

**iz4h4m2p** formed cocrystals with **dnba**, **2c4n** and **4nba**, yielding colourless plates, colourless blocks and colourless plates, respectively. Each cocrystal contains one molecule of **iz4h4m2p** and one molecule of the respective coformer in the asymmetric unit. Both **iz4h4m2p** + **2c4n** and **iz4h4m2p** + **4nba** crystallize in space group 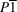 , while **iz4h4m2p** + **dnba** crystallizes in the space group *P*2_1_/*n*. It should be noted that the cocrystals of **iz4h4m2p** + **2c4n** and **iz4h4m2p** + **4nba** share similar unit-cell parameters and the same space group, which would imply the structures may be isostructural. Using the crystal structure similarity tool and restricting the size of the cluster to 30 molecules, there is a strong correlation that shows that the two structures may be isostructural to a degree, as seen in Fig. 5 ▸(*d*), despite some deviations observed (such as, for example, some of the pyridine ring having different torsion angles). This slight difference is due to the presence of the chlorine atom in **2c4n**. The major hydrogen bonding for all three structures is the same as the one described in the previously discussed structures: the carb­oxy­lic acid⋯pyridine ring hydrogen bond and the amide⋯hydroxyl⋯amide chain hydrogen bond. Nitro groups in all structures do not participate in any hydrogen bonding. π⋯π stacking exists between **2c4n** molecules to **2c4n** molecules and **iz4h4m2p** molecules to **iz4h4m2p** molecules in the structure of **iz4h4m2p + 2c4n** (**2c4n**⋯**2c4n**: *Cg*⋯*Cg* = 3.58 Å and **iz4h4m2p**⋯**iz4h4m2p**: *Cg*⋯*Cg* = 3.74 Å). A similar pattern of π⋯π stacking was observed for the structure of **iz4h4m2p** + **4nba** (**4nba**⋯**4nba**: *Cg*⋯*Cg* = 3.66 Å and **iz4h4m2p**⋯**iz4h4m2p**: *Cg*⋯*Cg* = 3.81 Å). For **iz4h4m2p** + **dnba,** the only π⋯π stacking observed was between adjacent **dnba** molecules (*Cg*⋯*Cg* = 3.90 Å). The packing of **iz4h4m2p** + **dnba**, **iz4h4m2p** + **2c4n** and **iz4h4m2p** + **4nba** is similar: alternating rows of 1D ribbons formed from the chain hydrogen-bond motif [Figs. 5 ▸(*a*)– ▸5(*c*)].

### 3.4. Crystal structure of **iz4h4m2p** + **4hba**

**Figure 6:**
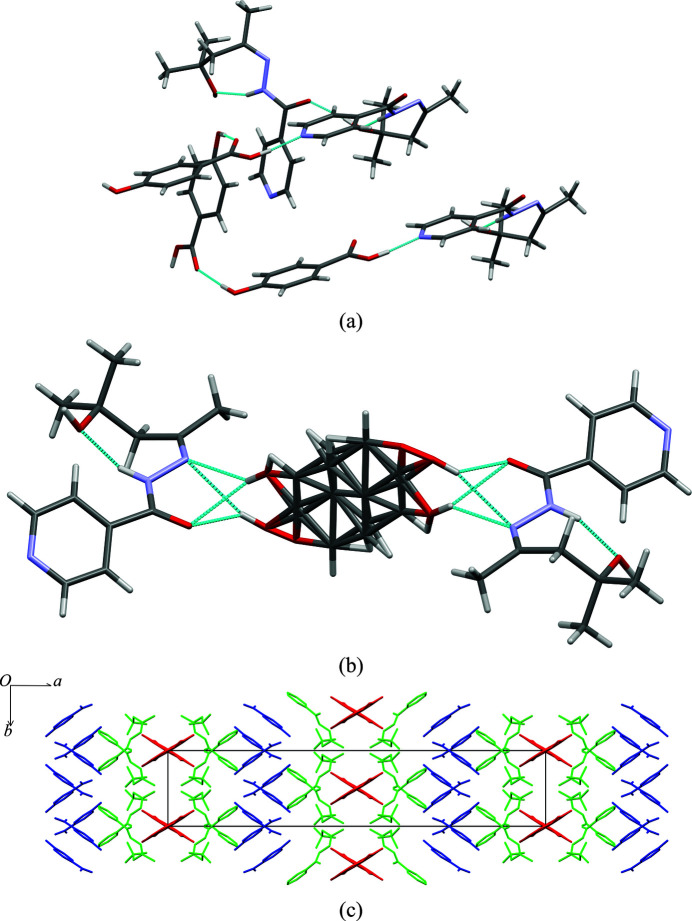
The crystal structure of **iz4h4m2p** + **4hba** showing (*a*) the hydrogen bonding between the non-disordered **4hba** molecules and **iz4h4m2p** (excluding the disordered **4hba** molecule), (*b*) the hydrogen bonding between the disordered **4hba** molecules and **iz4h4m2p** and (*c*) the packing of the structure down the *c*-axis [with hydrogens omitted for clarity; the molecules are colour-coded as follows: green: **iz4h4m2p**, blue: **4hba** (non-disordered) and red: **4hba** (disordered)].

**iz4h4m2p** and **4hba** formed a cocrystal, forming colourless plates. This cocrystal contains one molecule of **iz4h4m2p** and two molecules **4hba** in the asymmetric unit and crystallizes in space group *C*2/*c*. One of the two molecules of **4hba**, the one located at a special crystallographic site, is disordered. This causes the disordered **4hba** molecule to exist in one of two possible orientations at these sites. To distinguish between the two **4hba** molecules, they will be referred to as either the disordered or non-disordered **4hba** molecule. Not all the hydrogens attached to an oxygen atom could be found precisely and were placed geometrically with respect to the oxygen atom. These hydrogen atoms are H3, H6 and H8. Like the previous structures, the hydroxyl group of **iz4h4m2p** forms a hydrogen bond to the oxygen of the amide group from a neighbouring **iz4h4m2p** molecule (O2—H2⋯O1), while the carb­oxy­lic acid group of the non-disordered **4hba** molecule forms a hydrogen bond with the pyridine ring of **iz4h2m2p**. The hydroxyl group of the non-disordered **4hba** molecule forms a hydrogen bond with one of the oxygen atoms of the carb­oxy­lic acid group from a neighbouring **4hba** molecule (O5—H5*A*⋯O4) [Fig. 6 ▸(*a*)]. A π⋯π stacking interaction between the pyridine ring of **iz4h4m2p** and the aromatic ring of the non-disordered **4hba** molecule is present (*Cg*⋯*Cg* = 3.695 Å). Due to the nature of the varied orientation of the disordered **4hba** molecule and it sitting on an inversion site, two sets of almost identical hydrogen bonding are present. The hydrogen atoms from the carb­oxy­lic acid group (O6—H6) and the hydroxyl group (O8—H8) to the one nitro­gen atom of the hydrazine group (N3) and the oxygen atom of the amide (O1). The packing is made up of a series of alternating layers of the disordered **4hba** molecules, **iz4h4m2p** molecules and non-disordered **4hba** molecules.

### 3.5. Crystal structures of **iz4h4m2p** + **2,5-dhba** and **iz4h4m2p** + **3hba**

**Figure 7:**
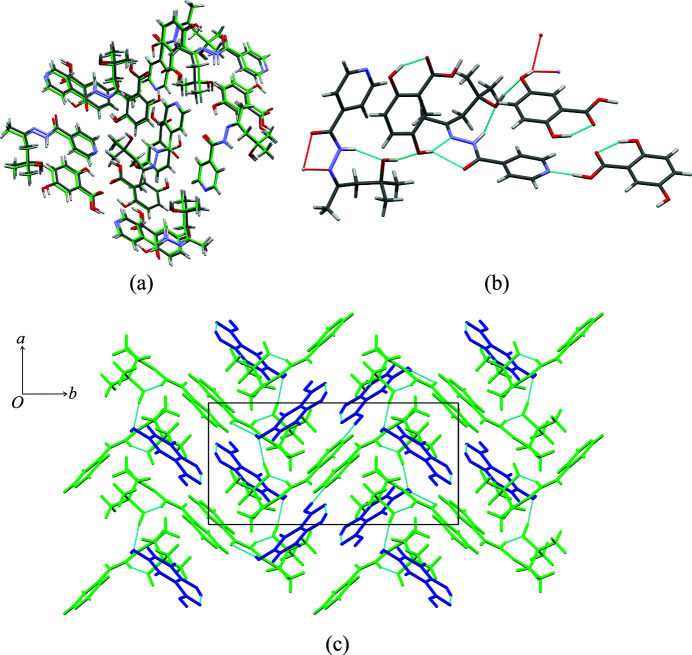
The crystal structure of **iz4h4m2p** + **2,5-dhba** showing (*a*) the overlap of **iz4h4m2p** + **3hba** and **iz4h4m2p** + **2,5-dhba** using the crystal structure similarity tool in *MERCURY* (**iz4h4m2p** + **3hba** in standard colours while the carbon atoms in **iz4h4m2p + 2,5-dhba** are in green), (*b*) the hydrogen bonding present and (*c*) the packing diagram (**iz4h4m2p** is presented in green while **2,5-dhba** is presented in blue).

**iz4h4m2p** formed cocrystals with **2,5-dhba** and **3hba,** forming colourless blocks and colourless plates, respectively. The asymmetric unit for both structures consists of one molecule of each **iz4h4m2p** and the respective coformer, forming in space group *P*2_1_/*n*. In our initial refinement of the crystal structure for **iz4h4m2p** + **2,5-dhba** we observed some electron density that located around one of the inversion sites. It is plausible that this electron density could correlate to some methanol molecules partially occupying these sites. However, this electron density was determined to be too weak to be refined as proper atoms, therefore we used the *SQUEEZE* program of *PLATON* to remove it (Spek, 2020 ▸). In addition, the hydrogen atom H3 in **iz4h4m2p** + **2,5-dhba** could not be found precisely, and was placed geometrically with respect to the O3 atom. Due to both structures having similar unit-cell parameters, the crystal structure similarity tool in *MERCURY* was used to confirm whether both structures are isostructural. The search was restricted to a cluster of 15 molecules and molecular differences were allowed. From Fig. 7 ▸(*a*) it can be seen that all 15 molecules matched, which is indicative of both structures being isostructural. This should not be too surprising, since the difference between **3hba** and **2,5-dhba** is simply the additional hydroxyl group on **2,5-dhba**, which ultimately forms an intramolecular hydrogen bond with the carb­oxy­lic acid group. Both structures are also unusual with respect to most of the other cocrystals presented here, as the amide⋯hydroxyl⋯amide (**iz4h4m2p**) chain hydrogen-bond motif does not exist. Instead, the hydroxyl group of the carb­oxy­lic acid forms a bifurcated hydrogen bond with the amide and imine group’s nitro­gen (O5—H5*A*⋯O1 and O5—H5*A*⋯N3 for **iz4h4m2p** + **3hba** and O6—H6⋯N3 and O6—H6⋯O1 for **iz4h4m2p** + **2,5-dhba**). The hydroxyl group of **iz4h4m2p** then forms a hydrogen bond to the phenol of the benzoic acid (in place of the amide group from a neighbouring **iz4h4m2p** molecule). This, in turn, forms a new chain hydrogen-bond motif 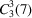 , which alternates between **iz4h4m2p** and the respective benzoic acid molecules [Fig. 7 ▸(*b*)]. Due to this chain hydrogen-bond motif, the packing of **iz4h4m2p** + **2,5-dhba** forms a series of zigzag ribbons [Fig. 7 ▸(*c*)].

### 3.6. Comparison of cocrystals with literature examples

The CSD was used to evaluate and compare the cocrystals presented in this work with other cocrystals containing **inh** (or a Schiff-based derivative of **inh**). A complete list of CSD entries with their refcodes used to make these comparisons is included in the supporting information. First, for cocrystals and molecular salts containing **inh**, 78 results were obtained after removing duplicate entries, alkali or alkali metal salts, inorganic acids and other derivatives of **inh**. Almost all cocrystals of **inh** were formed using a coformer with a carb­oxy­lic acid functional group, forming either a carb­oxy­lic acid⋯­pyridine ring hydrogen bond or a proton transfer if the result was a molecular salt. Nine cocrystals containing a coformer without a carb­oxy­lic were found which include the following: hydro­chloro­thia­zide (CSD refcode: DADLUS), cyanuric acid (CSD refcode: MOXNAR), 2,4,6-tri­nitro­phenolate (CSD refcode: PEZVAU), 5-fluoro­cytosine, (CSD refcode: PINJII), 5,7-di­hydroxy-2-(4-hy­droxy­phenyl)-2,3-di­hydro-4*H*-1-benzo­pyran-4-one (CSD refcode: UDUJIP), 1,5-naphthalenedi­sulfonic acid (CSD refcode: VEGHUN), resveratrol (CSD refcode: VOPQEZ), naphthalene-2,7-diol (CSD refcode: KEBLUC) and nitro­furan­toin monohydrate (CSD refcode: KEFXOM). The CSD contains 433 hits of Schiff-based **inh** derivatives with the imine functional group, of which 227 contain more than one chemical entity (molecules, ions *etc*.), of which 131 were hydrates. After removing inorganic salts and solvates, only 53 cocrystals and molecular salts containing a Schiff-based **inh**-derivative remained. Like **inh**, most of the coformers used to synthesize these cocrystals and molecular salts contains a carb­oxy­lic acid functional group. Exceptions include 2,4,6-tri­nitro­phenolate (CSD refcodes: CETWEF, EPISAY, LIGXEG and ZECXEN), saccharin (CSD refcodes: ZUVWUK and ZUVXAR) and acesulfame (CSD refcode: OKAVUT).

Similar to the cocrystals of **iz4h4m2p** presented here, cocrystals featuring a carb­oxy­lic acid will form a carb­oxy­lic acid⋯pyridine ring hydrogen-bond motif. A large degree of variability in hydrogen bonding exists in cocrystals containing **inh**. Cocrystals featuring an isoniazid derivative have a limited degree of variability in the hydrogen bonding, most of which stems from the nature of the coformer chosen. The variability of hydrogen bonding in **inh** cocrystals can include forming the following hydrogen-bond motifs: homodimers between the hydrazide groups, tetramers and chains. The packing can vary drastically in crystals containing **inh** molecules, which can range from 1D ribbons to 3D networks, which is due to the different combinations of hydrogen-bond pairing. For the cocrystals containing an isoniazid derivative with a reduced number of hydrogen pair donors, most packing is reduced to 1D ribbons, which can stack either parallel (such as **iz4h4m2p** + **dnba**, **iz4h4m2p** + **2c4n** and **iz4h4m2p** + **4nba**) or zigzag (such as **iz4h4m2p** + **2,5-dhba** and **iz4h4m2p** + **sa**).

Some exceptional cases arise. For example, in the cocrystal of the isoniazid derivative derivatized using acetone [*N*′-(propan-2-yl­idene)isonicotinohydrazide] and **2c4n** (CSD refcode: LATLID) (Lemmerer, 2012 ▸) contains both neutral and ionized species in the same asymmetric unit. Not only is this crystal structure chiral, the pyridine rings alternate in alignment along the chain hydrogen-bond motif at different points, which has not been observed in any of the cocrystals of **iz4h4m2p**. Polymorphism exists for the case of **inh** and cinammic acid, where three different forms exist (CSD refcodes: SETSAN01, SETSAN02, SETSAN03) (Sarcevica *et al.*, 2013 ▸, 2014 ▸). All three polymorphs featured forming an 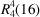 ring-based tetramer, connected using the carb­oxy­lic acid⋯pyridine hydrogen pair as well as one of the hydrazide hydrogens forming a hydrogen pair to the oxygen of the carb­oxy­lic acid, similar to the one reported for **inh** + **dnba**. Form **I** and form **II** exhibit the same hydrogen bonding pattern, which forms tetramers. The difference between form **I** and form **II** is the orientation in which the tetramers exist in relation to each other: the tetramers in form **I** are parallel while in form **II** some of the tetramers lie at an angle. Form **III** has different hydrogen bonding compared to the form **I** and form **II**, which features a bifurcated hydrogen bond between the one oxygen of the carb­oxy­lic acid to two hydrogens from the hydrazide group from different **inh** molecules. The packing of form **III** is similar to form **I**, a layered packing. In comparison, **iz4h4m2p** + **ca** forms a zigzag packing. This is a clear example that derivatizing **inh** using a Schiff-base reaction and reducing the number of hydrogen bond donors can reduce the complexity of a structure to a simpler one.

### 3.7. FTIR spectra

**Figure 8:**
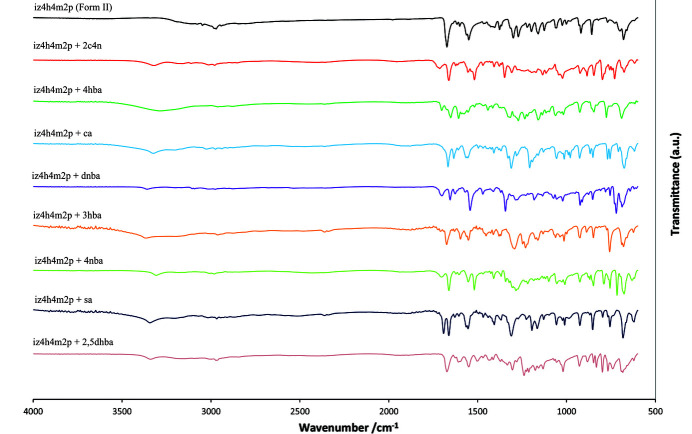
FTIR spectra of **iz4h4m2p** (form **II**) and its cocrystals.

FTIR spectroscopy is a useful technique to identify the different functional groups present in a solid, as well as to measure the strength of the intermolecular interactions between the different functional groups. For cocrystals, it is expected that FTIR spectra will differ to the spectra of the individual components, not just due to the addition of potential new functional groups but also due to the different intermolecular interactions occurring (*i.e.* new hydrogen bonds *etc*.). In particular, hydrogen bonds will cause a shift of significant FTIR peaks, which will be dependent on the strength or degree of the hydrogen bond in question. The FTIR spectra are represented together in Fig. 8 ▸, while each spectra is presented separately in the supporting information. The spectral assignments for the carb­oxy­lic acid functional group (or for **iz4h4m2p** the hydroxyl and carbonyl groups) for the cocrystals and both forms of **iz4h4m2p** have been included in Table 3 ▸. Another important use of FTIR here is to determine whether proton transfer occurred or not in the cocrystal of **iz4h4m2p** + **2,5-dhba**. As noted before, the acidic protons from **2,5-dhba** could not be located precisely in the difference Fourier maps, which were assigned geometrically with respect to the carb­oxy­lic acid. FTIR can be used to determine whether proton transfer has occurred or not. To determine whether a proton transfer has occurred, one could check to see if stretching band of either the carbonyl group (C=O) or the hydroxyl group (O—H) is present or absent. If these characteristic peaks are absent, a salt would be present. Unfortunately **iz4h4m2p** has both a carbonyl (associated with the amide) and hydroxyl groups present, making identification of a salt using this method difficult. However, a carboxyl­ate group can also be identified by strong peaks associated with the symmetric and asymmetric stretching of the CO_2_^−^ group in the 1450–1360 cm^−1^ and 1650–1540 cm^−1^ ranges, respectively. With the exception of **iz4h4m2p** + **dnba** these peaks are absent in each spectrum, indicating that no proton transfer occurs. For the case of **iz4h4m2p** + **dnba**, the asymmetric stretching of the nitro groups occurs in the same range of the asymmetric stretching of the CO_2_^−^ group, making this identification elusive. However, based on the absent strong CO_2_^−^ group symmetric stretching band, it is more likely **iz4h4m2p** + **dnba** is a cocrystal as opposed to a salt.

### 3.8. Thermal analysis

**Figure 9:**
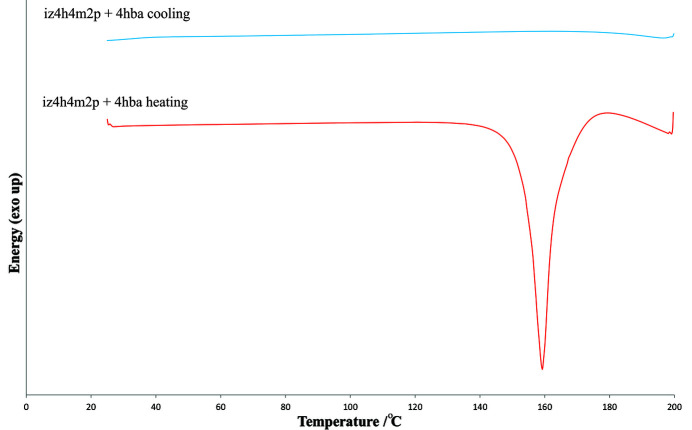
DSC diagrams of **iz4h4m2p** + **4hba** representing the typical behaviour of DSC curves of all cocrystals of **iz4h4m2p**.

DSC curves were collected for all eight cocrystals. Two of the DSC curves are presented in Fig. 9 ▸, with the curve of **iz4h4m2p** + **4hba** being a representative DSC curve for all the cocrystals. The DSCs of the other cocrystals are presented in the supporting information. The associated onset temperatures and enthalpies are represented in Table 4 ▸. For most of the samples, a single large endothermic peak was observed (corresponding to the melting/decomposition point of the sample) on the heating cycle, while no thermal events were observed on the cooling cycle which corresponds closely to the behaviour of form **II** of **iz4h4m2p**. It should be noted that only one large endothermic peak was observed for **iz4h4m2p** + **4hba**, which indicates that desolvation does not occur before melting.

## 4. Conclusions

Eight cocrystals containing **iz4h4m2p** were synthesized and characterized. Most of the cocrystals were uniform with regard to their hydrogen bonding, with two major hydrogen-bonding motifs observed: the carb­oxy­lic acid⋯pyridine hydrogen bond and the chain hydrogen-bond motif formed between the amide and hydroxyl groups, which is the same hydrogen-bond motif observed in form **I**. Exceptions to this arose when the carb­oxy­lic acid coformer had additional hydrogen bond donors (the hy­droxy­benzoic acids), which caused the overall hydrogen-bonding patterns to deviate from the expected hydrogen-bonding motifs. Packing of the structures featured either a zigzag or a layered formation, which indicates a greater degree of predicting the supramolecular assimilation. Most cocrystals did not feature any additional thermal events in their DSC curves. The derivatization of **inh** with diacetone alcohol made the cocrystals of **iz4h4m2p** more predictable than the cocrystals formed with **inh**. This could potentially prove useful in the design and synthesis of new FDC drugs. As such it would prove useful to evaluate the biological activity of **iz4h4m2p** and its cocrystals with GRAS compounds, as well as stability studies in the presence of these other drugs.

## Supplementary Material

Crystal structure: contains datablock(s) iz4h4m2p2c4n, iz4h4m2p3hba, iz4h4m2pca, iz4h4m2pdnba, iz4h4m2psa, iz4h4m2p4nba, iz4h4m2p25dhba, 22mcsiz4h4m2p4hba. DOI: 10.1107/S2052520622009532/aw5075sup1.cif

Structure factors: contains datablock(s) iz4h4m2p2c4n. DOI: 10.1107/S2052520622009532/aw5075iz4h4m2p2c4nsup2.hkl

Structure factors: contains datablock(s) iz4h4m2p3hba. DOI: 10.1107/S2052520622009532/aw5075iz4h4m2p3hbasup3.hkl

Structure factors: contains datablock(s) iz4h4m2pca. DOI: 10.1107/S2052520622009532/aw5075iz4h4m2pcasup4.hkl

Structure factors: contains datablock(s) iz4h4m2pdnba. DOI: 10.1107/S2052520622009532/aw5075iz4h4m2pdnbasup5.hkl

Structure factors: contains datablock(s) iz4h4m2psa. DOI: 10.1107/S2052520622009532/aw5075iz4h4m2psasup6.hkl

Structure factors: contains datablock(s) iz4h4m2p4nba. DOI: 10.1107/S2052520622009532/aw5075iz4h4m2p4nbasup7.hkl

Structure factors: contains datablock(s) shelx. DOI: 10.1107/S2052520622009532/aw5075iz4h4m2p25dhbasup8.hkl

Structure factors: contains datablock(s) 22mcsiz4h4m2p4hba. DOI: 10.1107/S2052520622009532/aw507522mcsiz4h4m2p4hbasup9.hkl

Tables S1 and S2, Figs S1-S23. DOI: 10.1107/S2052520622009532/aw5075sup10.pdf

CCDC references: 2177244, 2177245, 2177246, 2177247, 2177248, 2177249, 2177250, 2177251

## Figures and Tables

**Table 1:** Coformers, molar ratios and solvent systems used to synthesize cocrystals presented in this work **3hba**: 3-hydroxybenzoic acid, **4hba**: 4-hydroxybenzoic acid, **2,5hba**: 2,5-dihydroxybenzoic acid, **2c4n**: 2-chloro-4-nitrobenzoic acid, **4nba**: 4-nitrobenzoic acid, **dnba**: 3,5-dinitrobenzoic acid, **ca**: cinammic acid, **sa**: succinic acid.

Coformer	Molar ratio (**iz4h4m2p**:coformer)	Solvent system
**3hba**	1:1	Ethanol
**4hba**	1:1	Ethanol
**2,5-dhba**	1:1	Methanol or 1:1 *v*/*v* methanol : aceto­nitrile
**2c4n**	1:1	Ethanol
**4nba**	1:1	Ethanol
**dnba**	1:1	Ethanol
**ca**	1:1	Methanol
**sa**	2:1	Ethanol

**Table:** For all structures: Mo *K*α radiation, Bruker APEX-II CCD diffractometer, multi-scan absorption correction using *SADABS* (Krause *et al.*, 2015 ▸). H atoms treated by a mixture of independent and constrained refinement.

	**iz4h4m2p** + **2c4n**	**iz4h4m2p** + **3hba**	**iz4h4m2p** + **ca**	**iz4h4m2p** + **dnba**
Crystal data				
Chemical formula	C_12_H_17_N_3_O_2_·C_7_H_4_NO_4_Cl	C_7_H_6_O_3_·C_12_H_17_N_3_O_2_	C_12_H_17_N_3_O_2_·C_9_H_8_O_2_	C_12_H_17_N_3_O_2_·C_7_H_4_N_2_O_6_
*M*_r_	436.85	373.4	383.44	447.41
Crystal system, space group	Triclinic, 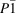	Monoclinic, *P*2_1_/*n*	Triclinic, 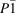	Monoclinic, *P*2_1_/*n*
Temperature (K)	173	173	173	173
*a*, *b*, *c* (Å)	7.0381 (1), 12.2643 (2), 12.4636 (2)	8.7472 (3), 16.9734 (5), 13.2480 (4)	6.9734 (3), 11.2540 (5), 13.4029 (6)	6.9538 (3), 18.9491 (8), 15.9042 (7)
α, β, γ (°)	103.215 (1), 97.797 (1), 93.497 (1)	90, 94.886 (1), 90	89.949 (2), 82.689 (2), 74.261 (2)	90, 99.261 (2), 90
*V* (Å^3^)	1032.92 (3)	1959.78 (11)	1003.55 (8)	2068.35 (15)
*Z*	2	4	2	4
μ (mm^−1^)	0.23	0.09	0.09	0.11
Crystal size (mm)	0.52 × 0.25 × 0.21	0.59 × 0.48 × 0.22	0.37 × 0.18 × 0.10	0.39 × 0.29 × 0.13

Data collection				
*T*_min_, *T*_max_	0.674, 0.746	0.686, 0.747	0.631, 0.746	0.699, 0.747
No. of measured, independent and observed *I* > 2σ(*I*)] reflections	49833, 7362, 4908	78332, 8649, 7062	47711, 4995, 4111	196011, 11136, 9039
*R*_int_	0.039	0.033	0.078	0.082
(sin θ/λ)_max_ (Å^−1^)	0.753	0.809	0.668	0.864

Refinement				
*R*[*F* ^2^ > 2σ(*F* ^2^)], *wR*(*F* ^2^), *S*	0.044, 0.117, 1.06	0.051, 0.145, 1.06	0.039, 0.107, 1.03	0.054, 0.140, 1.07
No. of reflections	7362	8649	4995	11136
No. of parameters	286	263	268	304
Δρ_max_, Δρ_min_ (e Å^−3^)	0.42, −0.35	0.50, −0.20	0.25, −0.20	0.54, −0.30

**Table:** 

	**iz4h4m2p** + **sa**	**iz4h4m2p** + **4nba**	**iz4h4m2p** + **25dhba**	**iz4h4m2p** + **4hba**
Crystal data				
Chemical formula	C_12_H_17_N_3_O_2_·C_2_H_3_O_2_	C_12_H_17_N_3_O_2_·C_7_H_5_NO_4_	C_12_H_17_N_3_O_2_·C_7_H_6_O_4_	C_12_H_16_N_3_O_2_·2(C_7_H_6_O_3_)
*M*_r_	294.33	402.4	389.4	884.92
Crystal system, space group	Monoclinic, *P*2_1_/*c*	Triclinic, 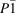	Monoclinic, *P*2_1_/*n*	Monoclinic, *C*2/*c*
Temperature (K)	173	173	133	173
*a*, *b*, *c* (Å)	6.9773 (4), 20.6482 (11), 10.5035 (5)	6.8701 (3), 12.1647 (6), 12.1779 (6)	8.5777 (5), 17.5407 (9), 13.2200 (8)	41.700 (2), 8.2992 (4), 12.9536 (8)
α, β, γ (°)	90, 98.764 (2), 90	101.130 (2), 92.818 (2), 105.005 (2)	90, 96.605 (2), 90	90, 97.033 (2), 90
*V* (Å^3^)	1495.56 (14)	959.26 (8)	1975.86 (19)	4449.2 (4)
*Z*	4	2	4	4
μ (mm^−1^)	0.10	0.11	0.10	0.09
Crystal size (mm)	0.52 × 0.23 × 0.07	0.57 × 0.42 × 0.11	0.58 × 0.49 × 0.27	0.55 × 0.26 × 0.14

Data collection				
*T*_min_, *T*_max_	0.676, 0.746	0.642, 0.746	0.689, 0.746	0.600, 0.746
No. of measured, independent and observed [*I* > 2σ(*I*)] reflections	45682, 3733, 3131	42678, 5857, 4742	41489, 4917, 4391	54732, 5529, 5529
*R*_int_	0.089	0.047	0.028	0.088
(sin θ/λ)_max_ (Å^−1^)	0.668	0.715	0.668	0.667

Refinement				
*R*[*F* ^2^ > 2σ(*F* ^2^)], *wR*(*F* ^2^), *S*	0.044, 0.113, 1.03	0.045, 0.136, 1.07	0.038, 0.105, 0.99	0.056, 0.150, 1.04
No. of reflections	3733	5857	4917	5529
No. of parameters	205	277	273	352
Δρ_max_, Δρ_min_ (e Å^−3^)	0.33, −0.21	0.34, −0.27	0.30, −0.21	0.29, −0.27

Computer programs: *SAINT* (v. 8.40B) (Bruker, 2016 ▸), *SHELXT-2018/2* (Sheldrick, 2015 ▸), *SHELXL2018/3* (Sheldrick, 2018 ▸), *ORTEP for Windows* (Farrugia, 2012 ▸), *Olex2* (v. 1.3) (Dolomanov *et al.*, 2009 ▸), *WinGX* publication routines (Farrugia, 2012 ▸).

**Table 3:** IR spectral assignments of the wavenumbers for **iz4h4m2p** and its cocrystals to the carb­oxy­lic acid functional and/or the hydroxyl and carbonyl functional groups

Sample	C=O stretch (cm^− 1^)	O—H stretch (cm^−1^)	C—O stretch (cm^−1^)	O—H bend in-plane (cm^−1^)	O—H bend out-plane (cm^−1^)
**i4h4m2p** (form **I**)	1660	2969	1273	1407	932
**i4h4m2p** (form **II**)	1673	2971	1271	1404	919
**iz4h4m2p** + **2c4n**	1662	2980	1309	1408	924
**iz4h4m2p** + **3hba**	1674	2964	1292	1412	924
**iz4h4m2p** + **4nba**	1662	2982	1285	1410	925
**iz4h4m2p** + **ca**	1667	2975	1281	1409	927
**iz4h4m2p** + **sa**	1662	2968	1309	1407	925
**iz4h4m2p** + **dnba**	1655	2976	1286	1412	912
**iz4h4m2p** + **4hba**	1651	2962	1271	1415	926
**iz4h4m2p** + **2,5-dhba**	1673	2972	1305	1413	928

**Table 4:** Onset temperatures and associated enthalpies for thermal events observed in DSC curves for the cocrystals presented in this work The melting/decomposition points of both polymorphic forms of **iz4h4m2p** are also given for comparison purposes (Scheepers *et al.*, 2022 ▸).

Thermal event	Onset (°C)	Enthalpy (J g^−1^)
**iz4h4m2p** (form **I**) melting/decomposition	139.7 ± 0.1	137.2 ± 2.3
**iz4h4m2p** (form **II**) melting/decomposition	142.1 ± 0.1	144.2 ± 2.4
**iz4h4m2p** + **2c4n** melting/decomposition	117.1 ± 0.2	237.7 ± 2.3
**iz4h4m2p** + **3hba** melting/decomposition	145.6 ± 0.2	276.8 ± 4.3
**iz4h4m2p** + **4nba** melting/decomposition	155.8 ± 0.2	279.9 ± 5.2
**iz4h4m2p** + **ca** melting/decomposition	103.4 ± 0.1	102.3 ± 6.6
**iz4h4m2p** + **dnba** melting/decomposition	130.5 ± 0.2	263.2 ± 2.8
**iz4h4m2p** + **sa** melting/decomposition	119.8 ± 0.1	102.2 ± 1.3
**iz4h4m2p** + **4hba** melting/decomposition	153.2 ± 0.2	233.9 ± 3.2
**iz4h4m2p** + **2,5-dhba** melting/decomposition	149.4 ± 0.2	300.6 ± 2.4
