# Supplementary material for: Design of a series of cocrystals featuring isoniazid modified with diacetone alcohol
Source: Acta Crystallogr B Struct Sci Cryst Eng Mater. 2022 Nov 8;78(Pt 6):857–67. doi: 10.1107/S2052520622009532 (PMC9728018; doi:10.1107/S2052520622009532)
Supplement: Supplementary file 10 [file b-78-00857-sup10.pdf]

STRUCTURAL SCIENCE
CRYSTAL ENGINEERING
MATERIALS

Volume 78 (2022)

Supporting information for article:

Design of a series of cocrystals featuring isoniazid modified with diacetone alcohol

Matthew Clarke Scheepers and Andreas Lemmerer

S1. Coformers used for this work

Table S1 Complete list of coformers used for this study.

Coformer	Mass used for a 1:1 stoichiometric amount relative to 50 mg of iz4h4m2p/ g
2-hydroxybenzoic acid (salicylic acid)	0.029
3-hydroxybenzoic acid	0.029
4-hydroxybenzoic acid	0.029
1-naphthionic acid	0.037
2-chloro-4-nitrobenzoic acid	0.043
2-chloro-5-nitrobenzoic acid	0.043
4-nitrobenzoic acid	0.036
3,5-dinitrobenzoic acid	0.045
Benzoic acid	0.026
2-aminobenzoic acid	0.029
3-aminobenzoic acid	0.029
4-aminobenzoic acid	0.029
Succinic acid	0.012
4-methoxybenzoic acid (methyl paraben)	0.032
trans-Cinnamic acid	0.031
Tartaric acid	0.032
2,4-dihydroxybenzoic acid	0.033
2,5-dihydroxybenzoic acid	0.033
3,4-dihydroxybenzoic acid	0.033
3,5-dihydroxybenzoic acid	0.033
Acetylsalicylic acid	0.038
Mendelic acid	0.032
Glycolic acid	0.008
2-chlorobenzoic acid	0.033
3-chlorobenzoic acid	0.033
4-chlorobenzoic acid	0.033
Toluic acid	0.029
1,4-dicarboxyliccyclohexane	0.037
4-iodobenzoic acid	0.053
4-bromobenzoic acid	0.043

S2. PXRD patterns

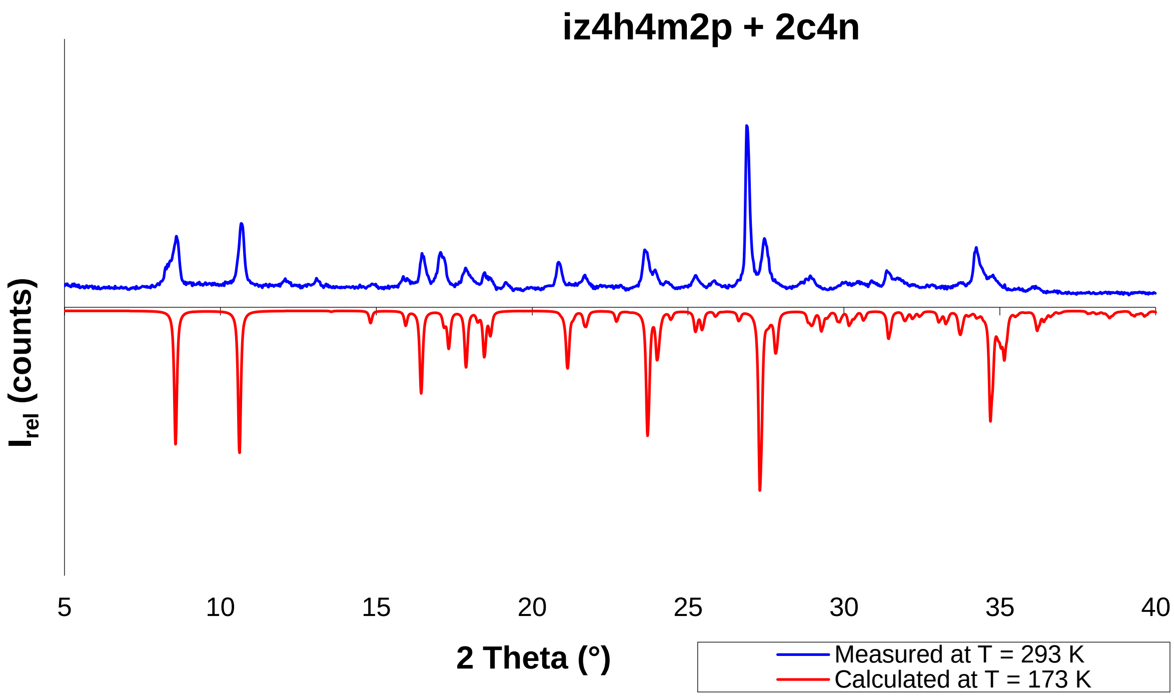

Figure S1 PXRD pattern for **iz4h4m2p** + **2c4n**

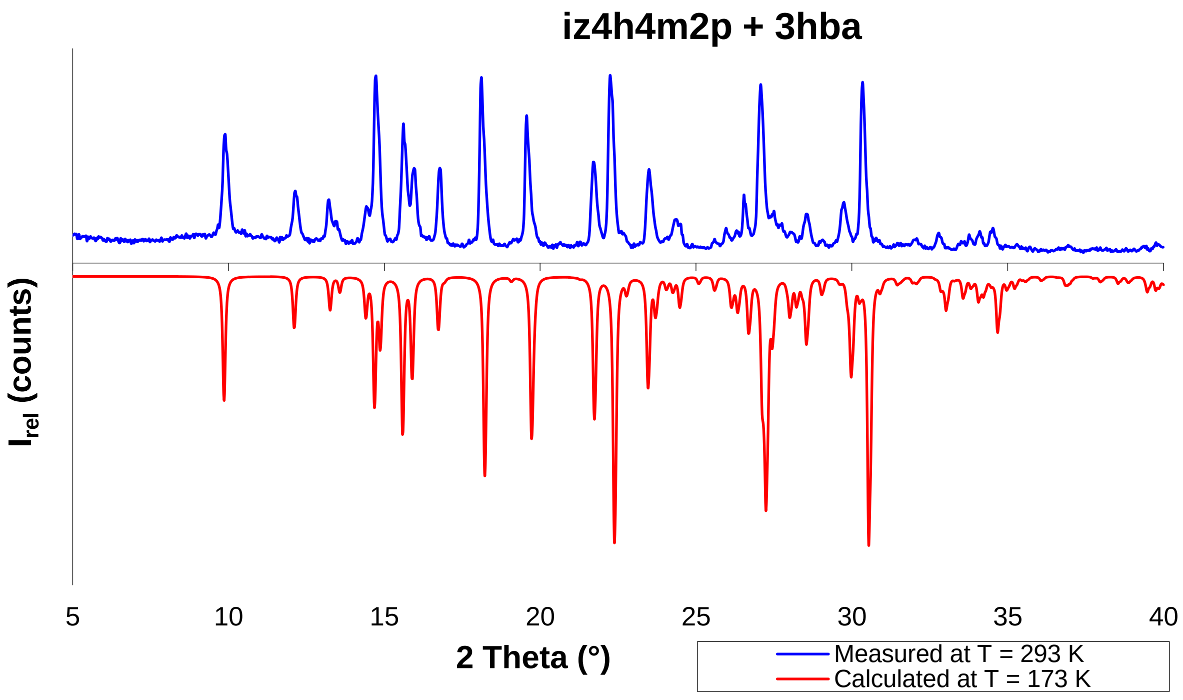

Figure S2 PXRD pattern for **iz4h4m2p** + **3hba**

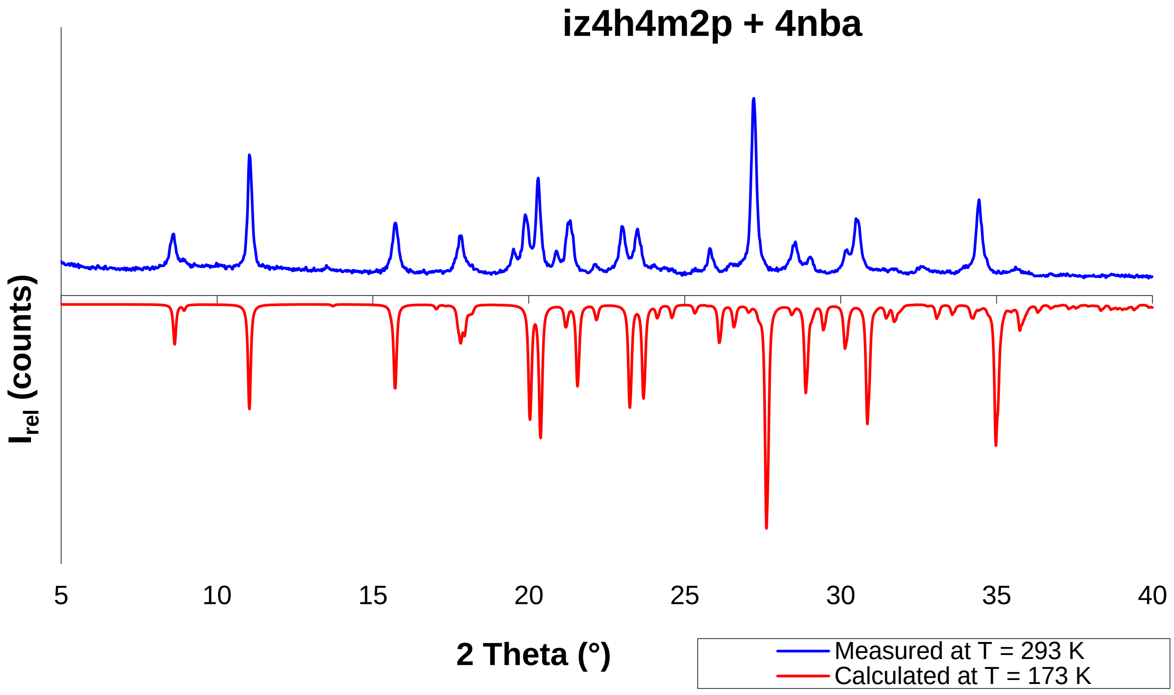

Figure S3 PXRD pattern for **iz4h4m2p** + **4nba**

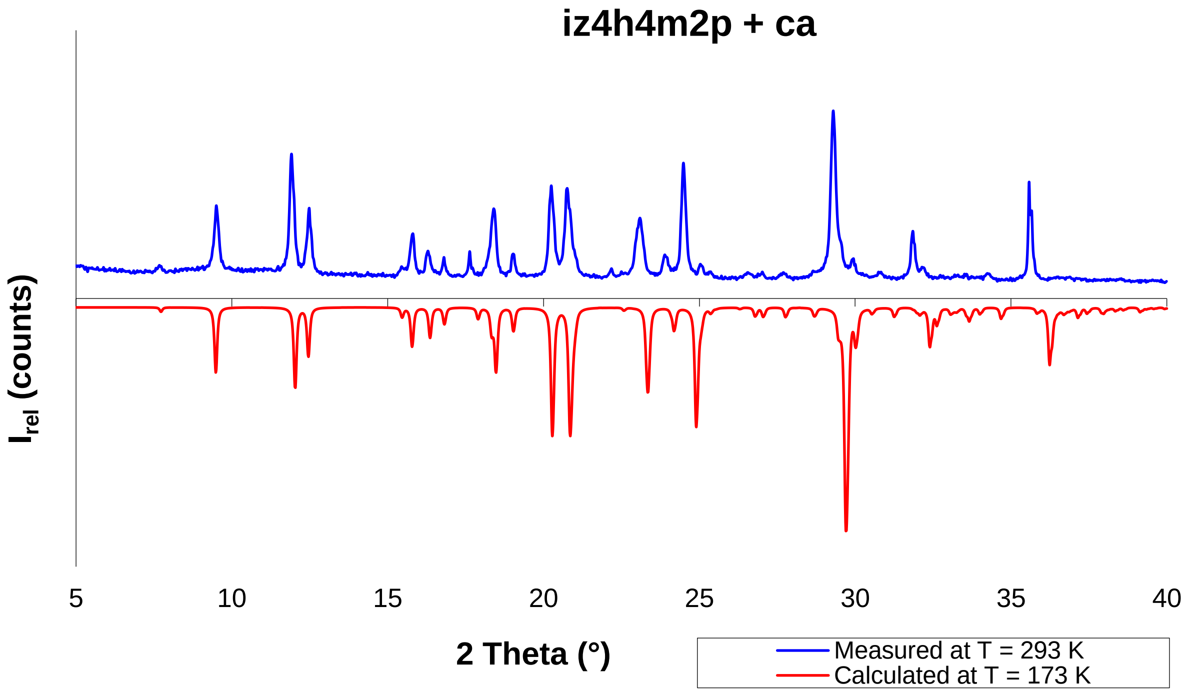

Figure S4 PXRD pattern for **iz4h4m2p** + ca

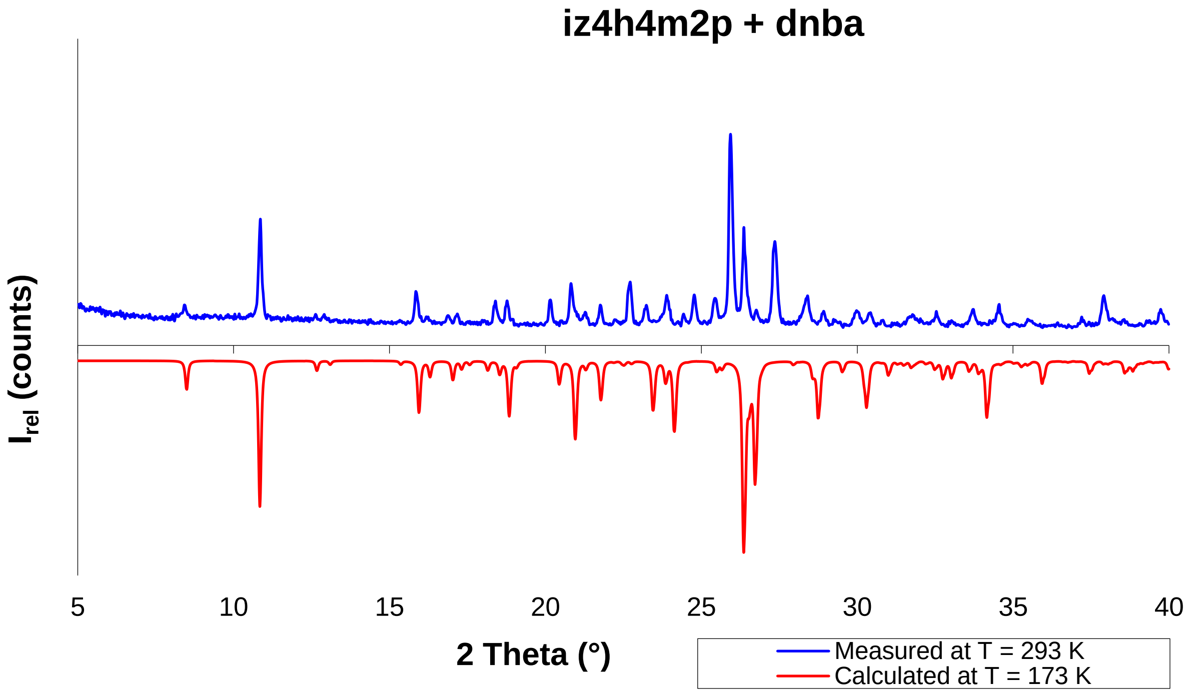

Figure S5 PXRD pattern for **iz4h4m2p** + **dnba**

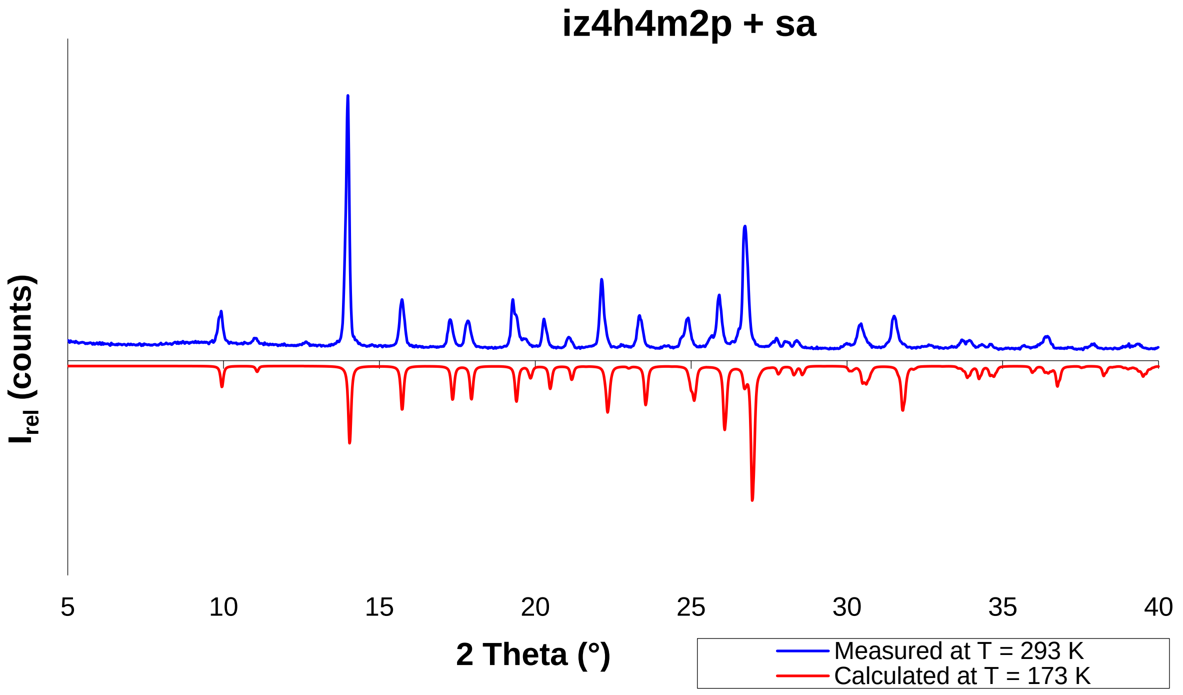

Figure S6 PXRD pattern for **iz4h4m2p** + sa

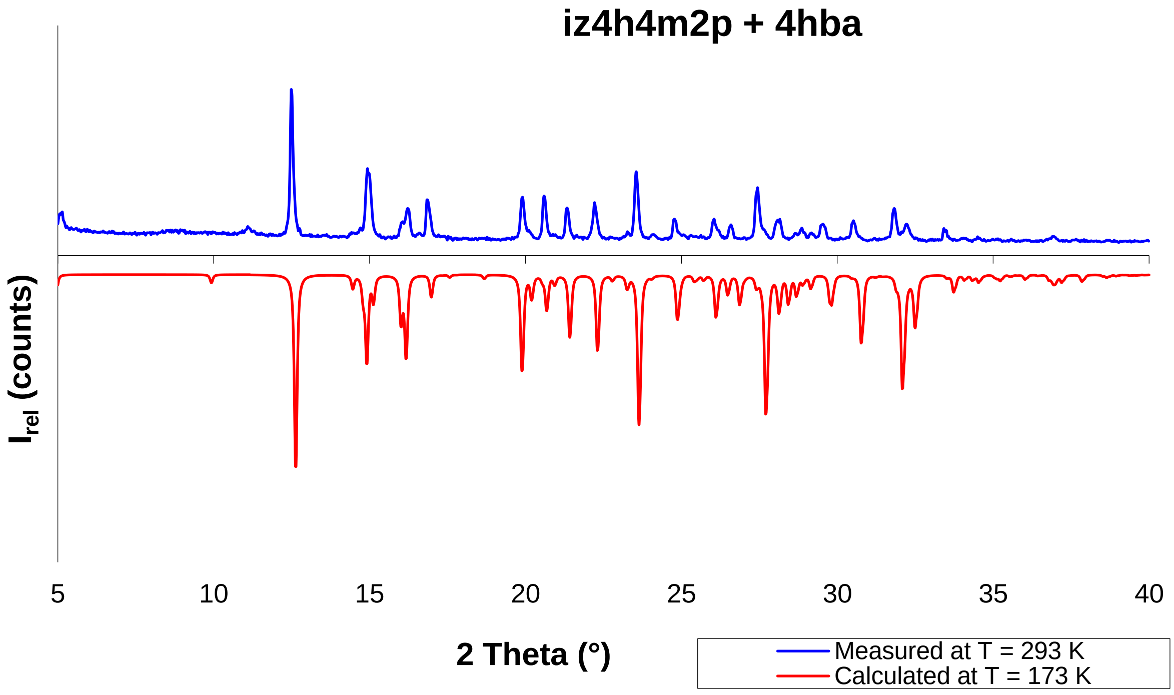

Figure S7 PXRD pattern for iz4h4m2p + 4hba

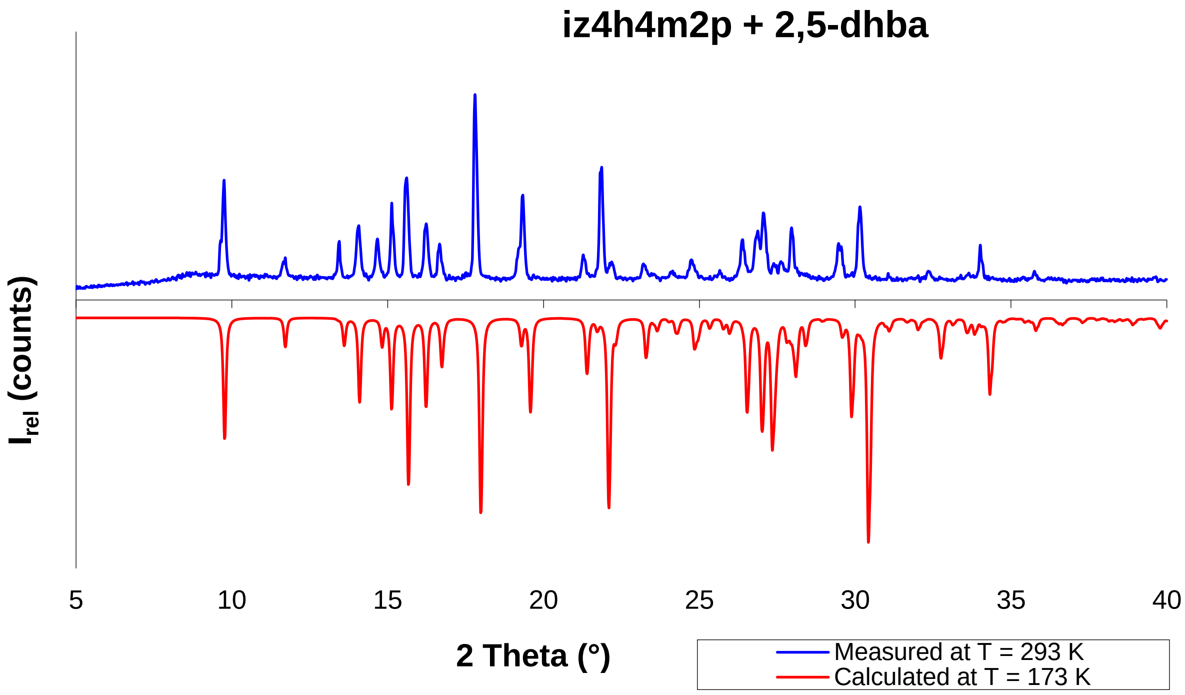

Figure S8 PXRD pattern for **iz4h4m2p** + **2,5-dhba**

S3. Individual FTIR spectra

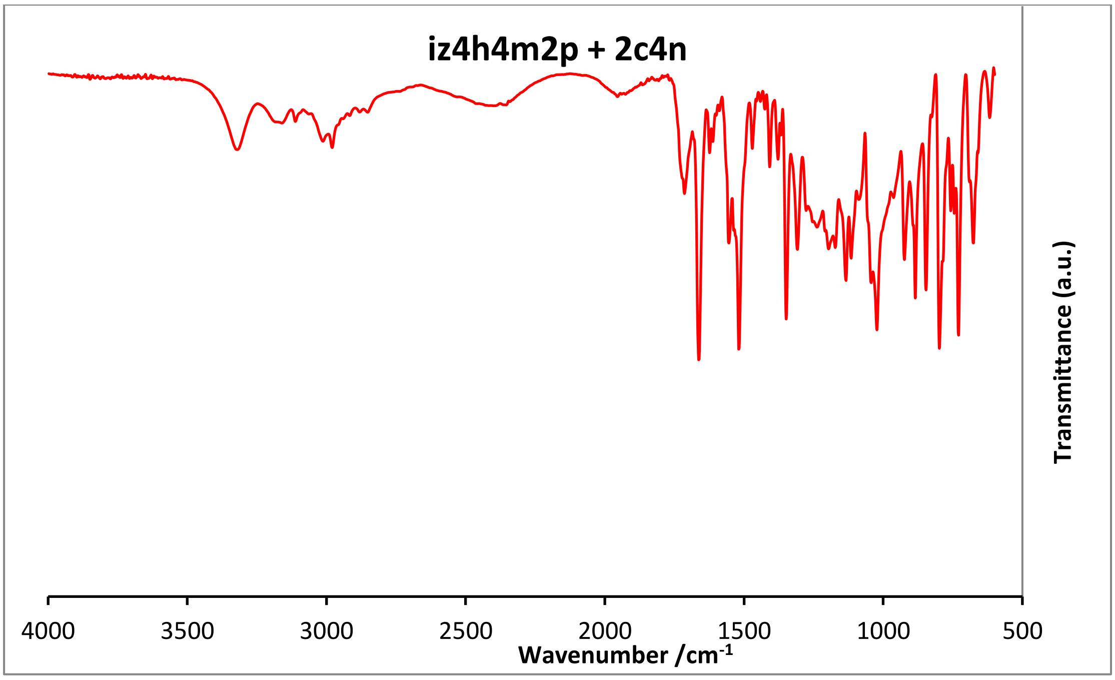

Figure S9 FTIR for the cocrystal of **iz4h4m2p** + **2c4n**

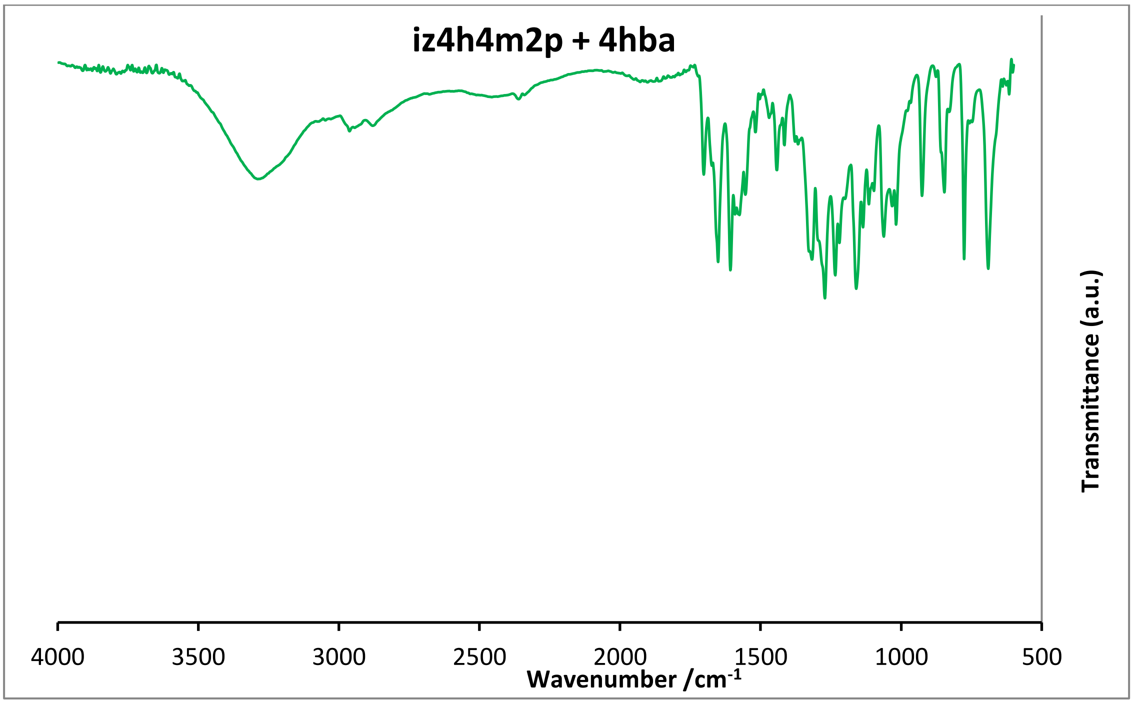

Figure S10 FTIR for the cocrystal of **iz4h4m2p** + **4hba**

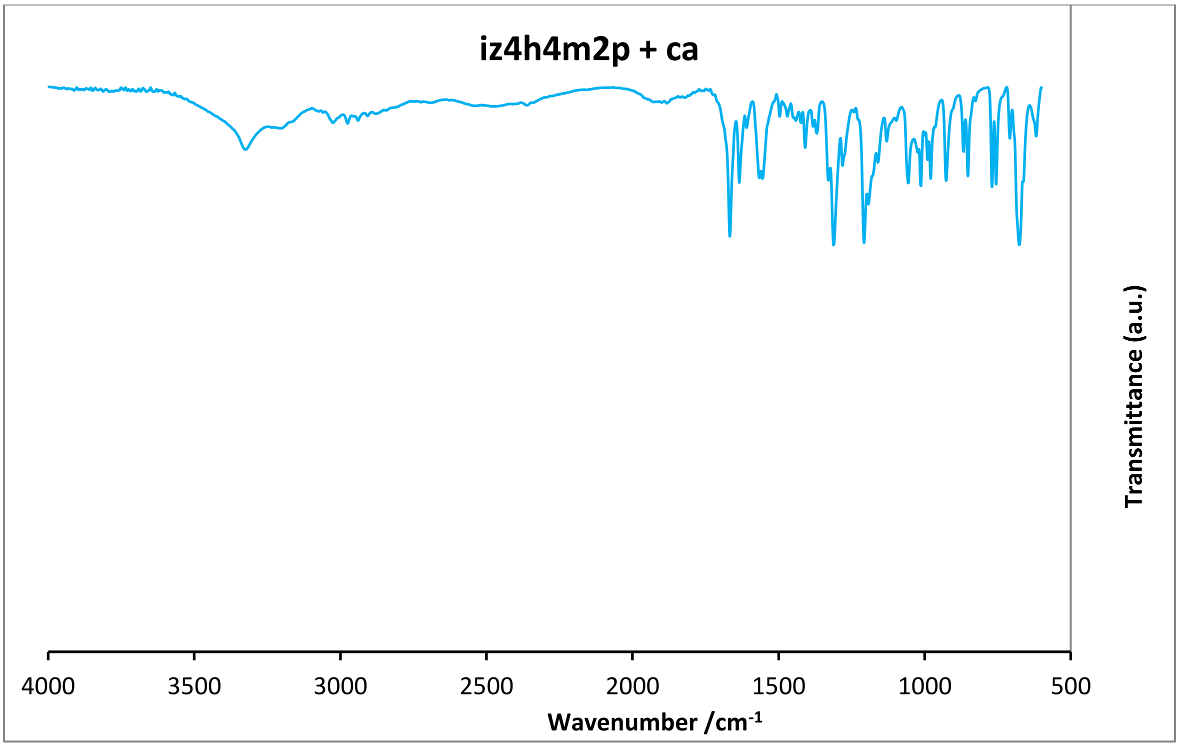

Figure S11 FTIR for the cocrystal of **iz4h4m2p** + **4hba**

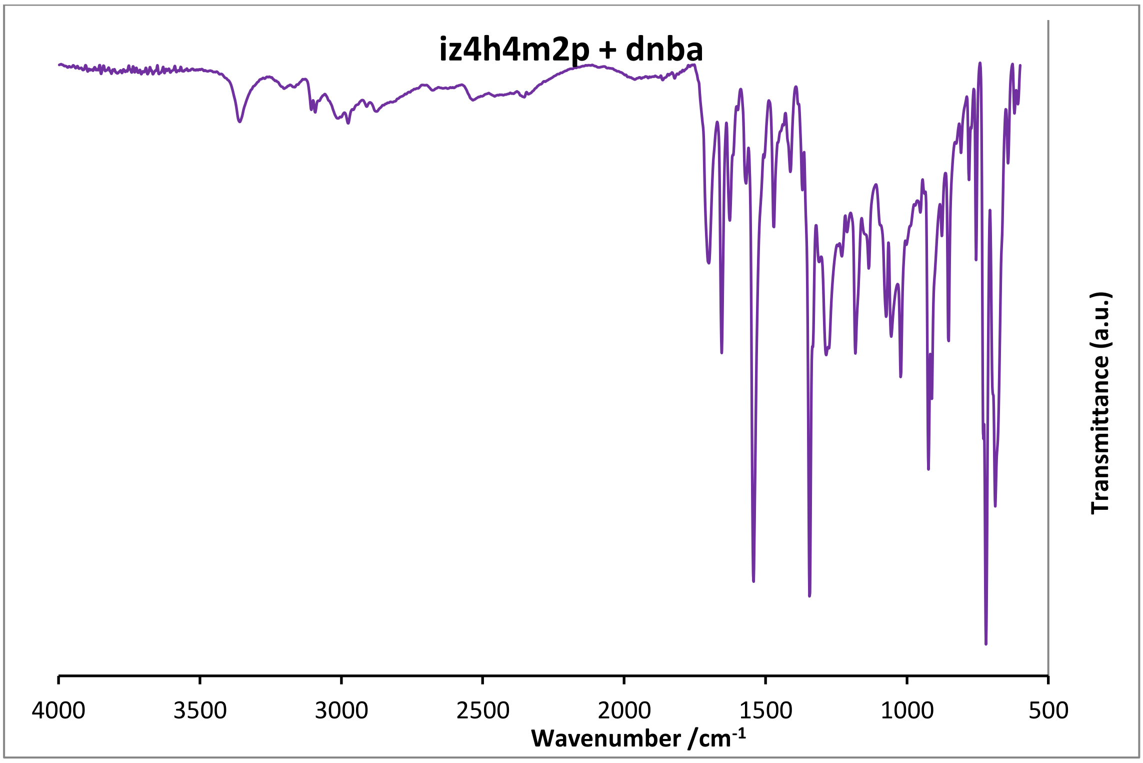

Figure S12 FTIR for the cocrystal of **iz4h4m2p** + **dnba**

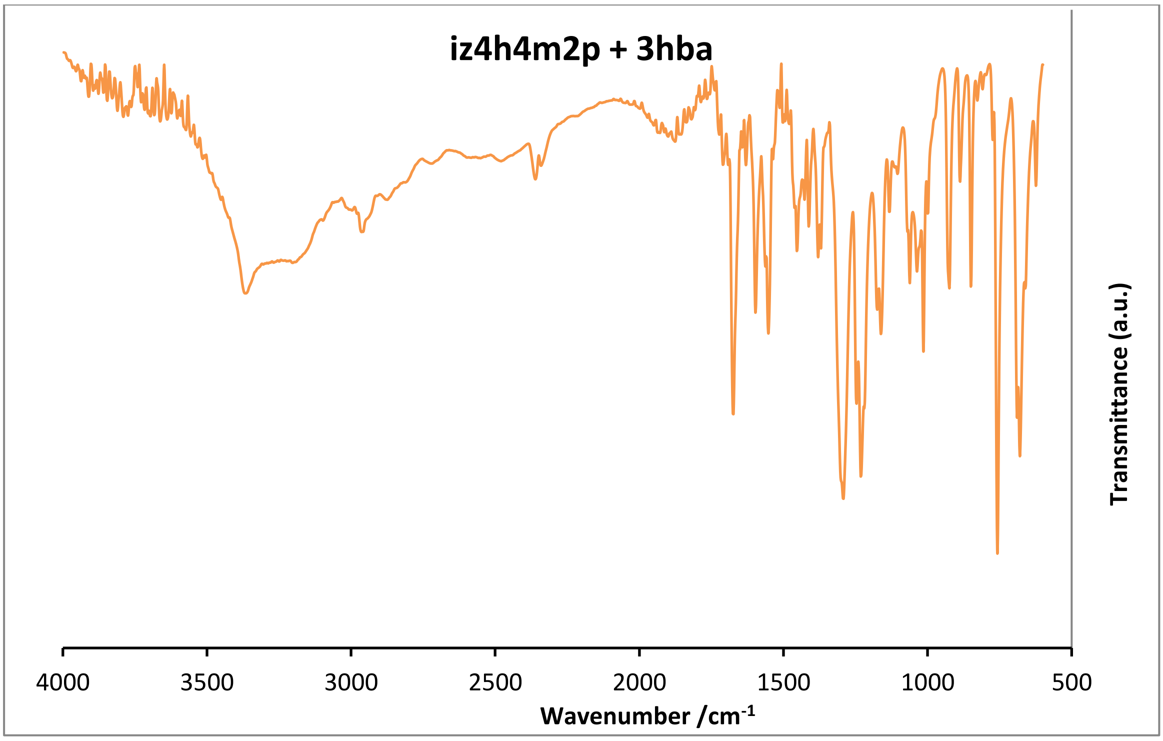

Figure S13 FTIR for the cocrystal of **iz4h4m2p** + **3hba**

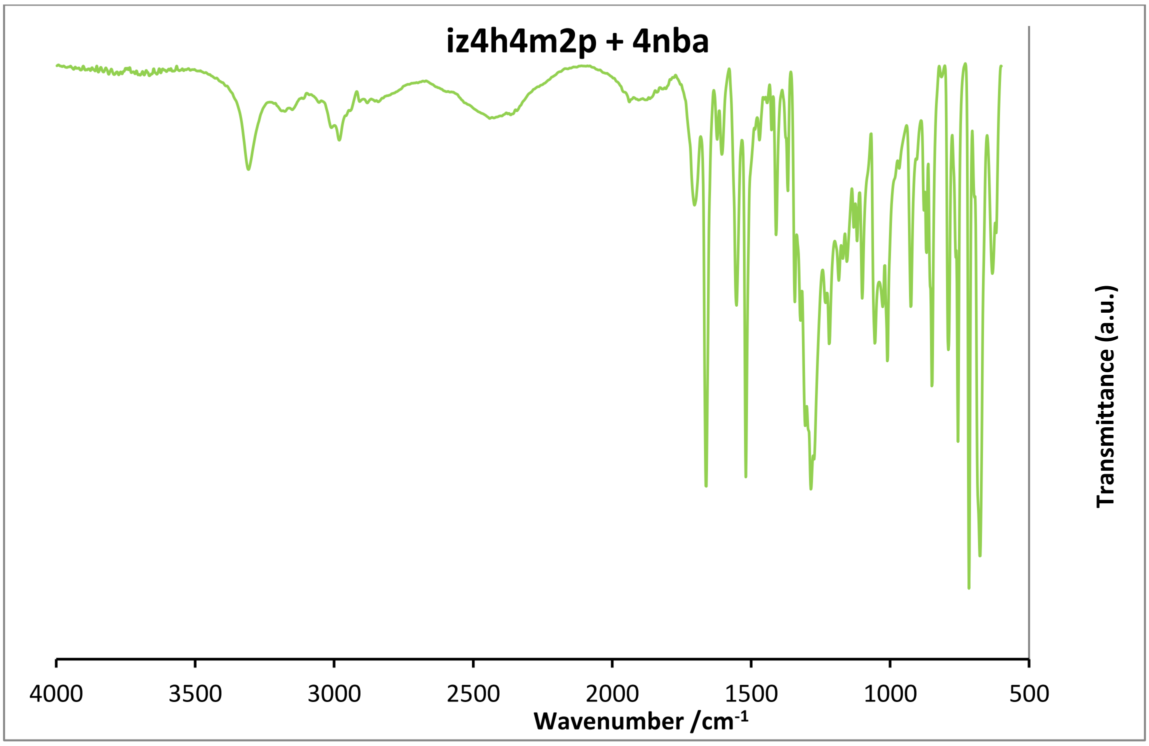

Figure S14 FTIR for the cocrystal of **iz4h4m2p** + **4nba**

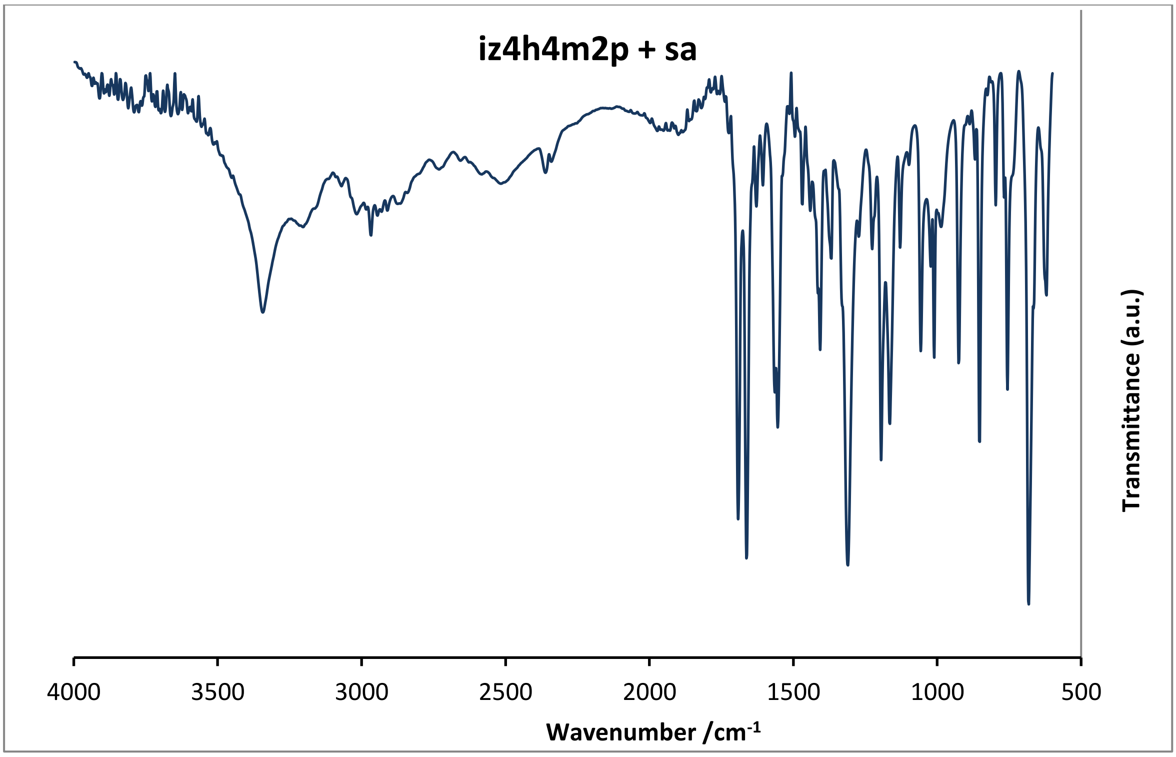

Figure S15 FTIR for the cocrystal of **iz4h4m2p** + **sa**

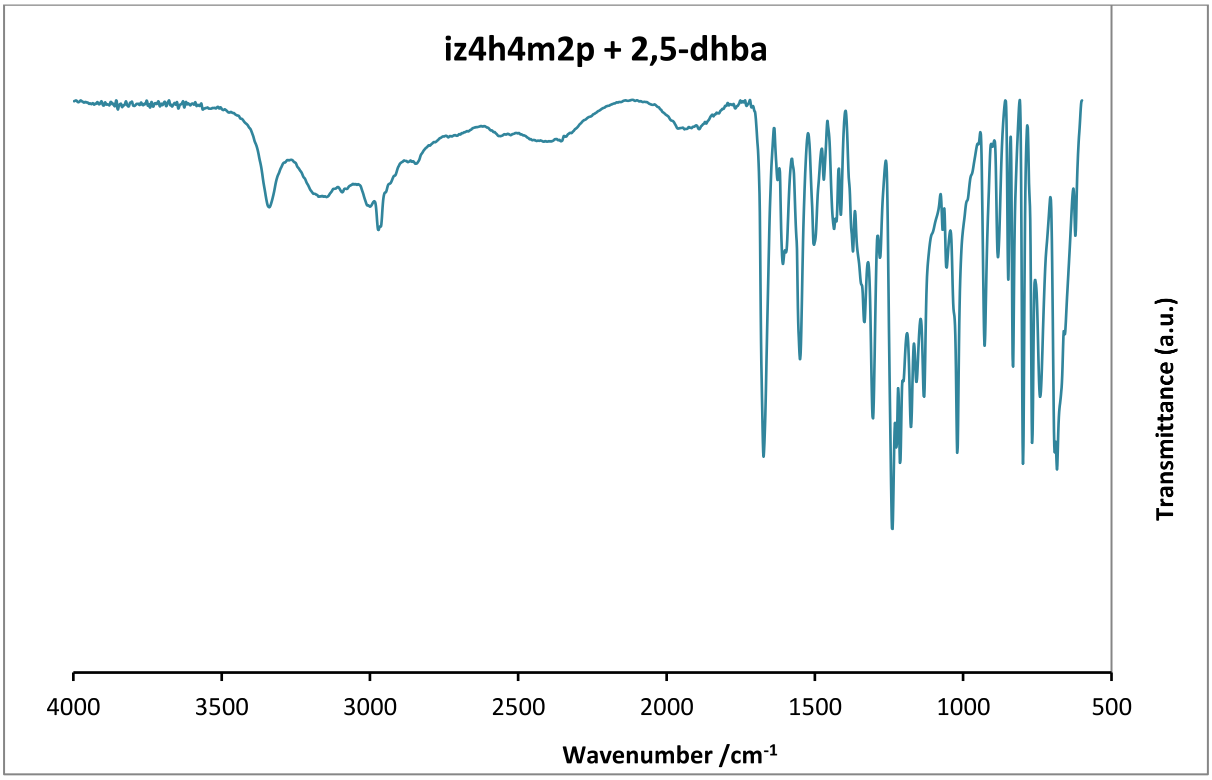

Figure S16 FTIR for the cocrystal of **iz4h4m2p** + **2,5-dhba**

S4. Individual DSC curves

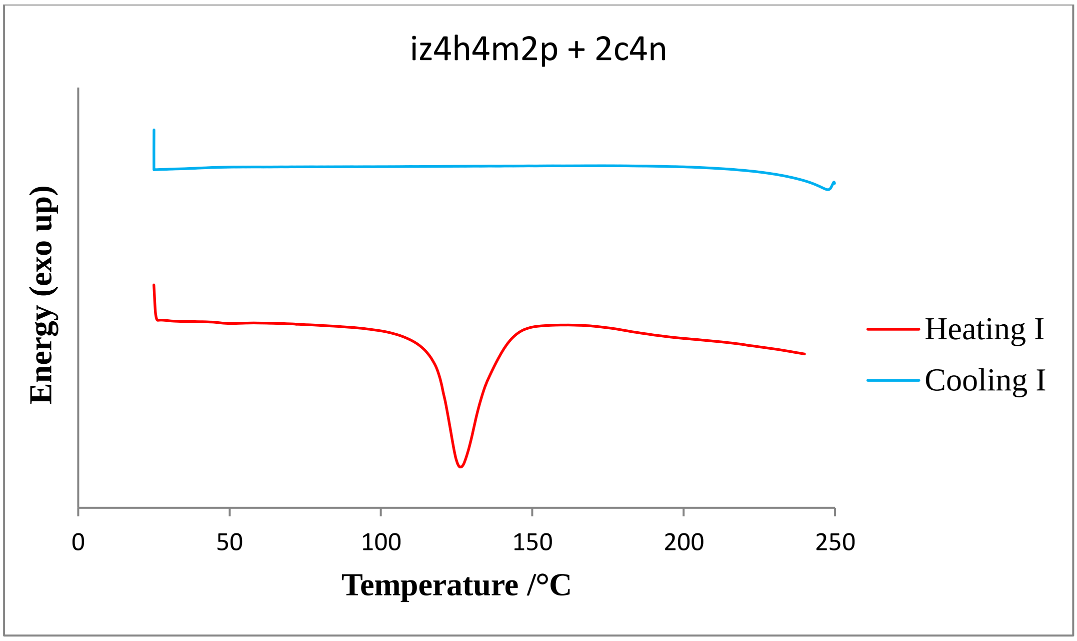

Figure S17 DSC curve for the cocrystal of **iz4h4m2p** + **2c4n**

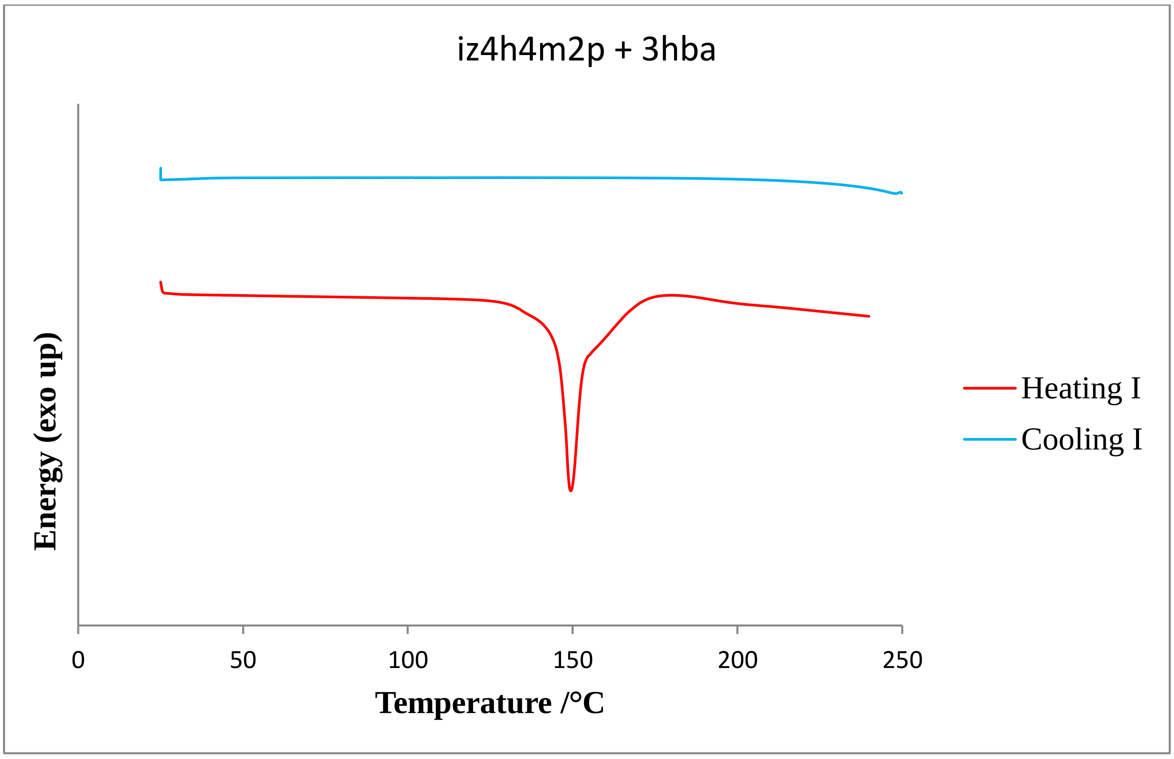

Figure S18 DSC curve for the cocrystal of **iz4h4m2p** + **3hba**

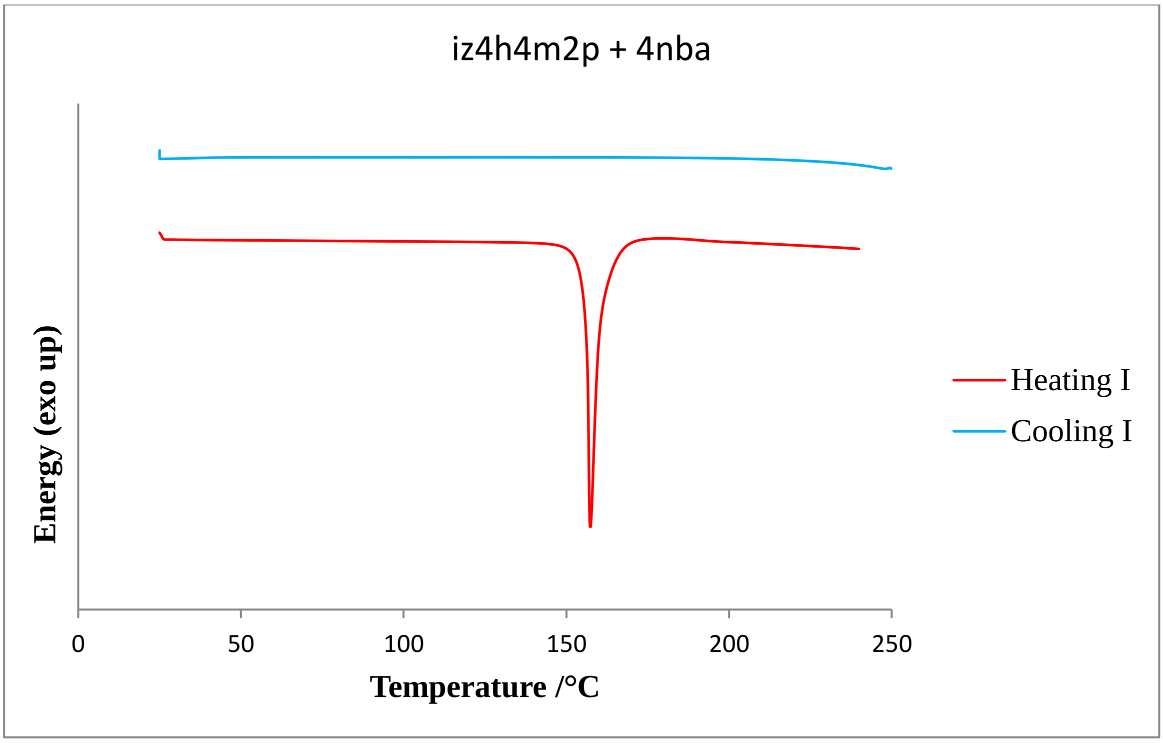

Figure S19 DSC curve for the cocrystal of **iz4h4m2p** + **4nba**.

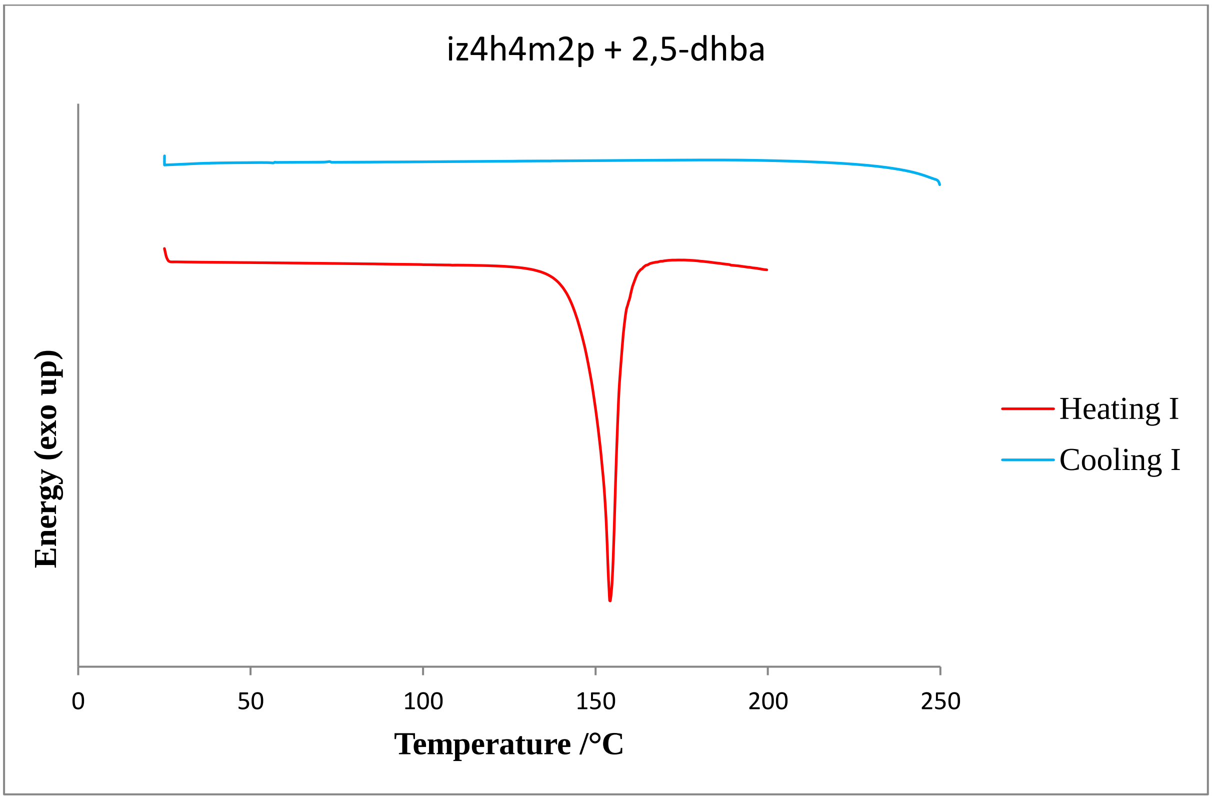

Figure S20 DSC curve for the cocrystal of **iz4h4m2p** + **2,5-dhba**.

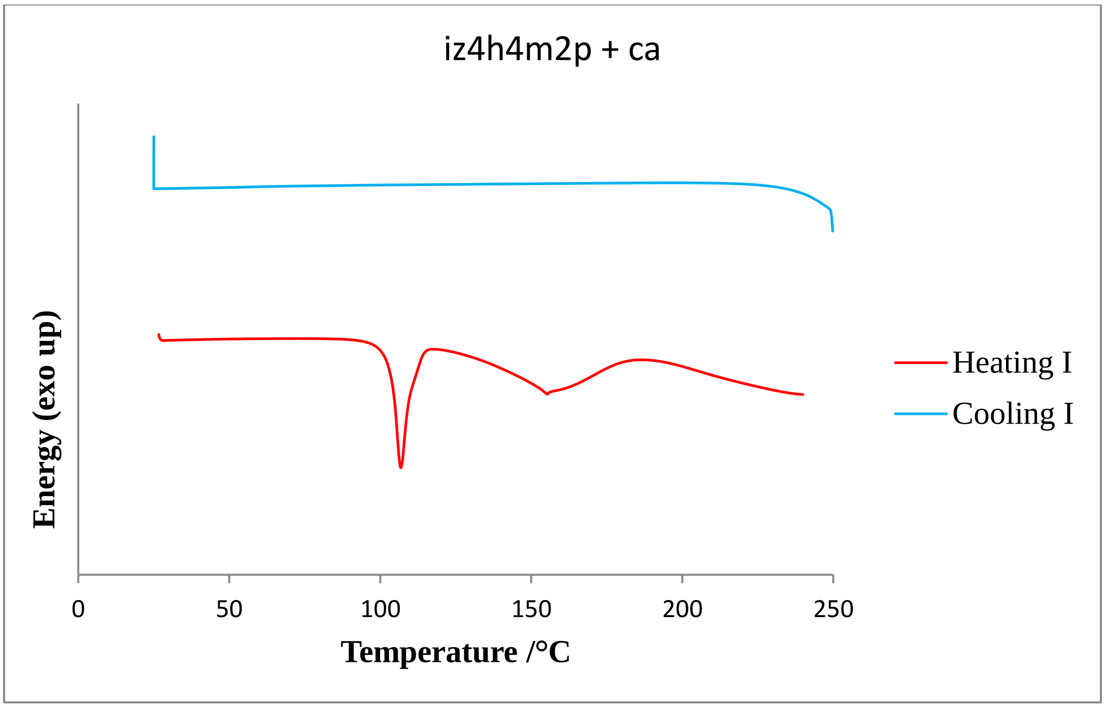

Figure S21 DSC curve for the cocrystal **iz4h4m2p** + ca

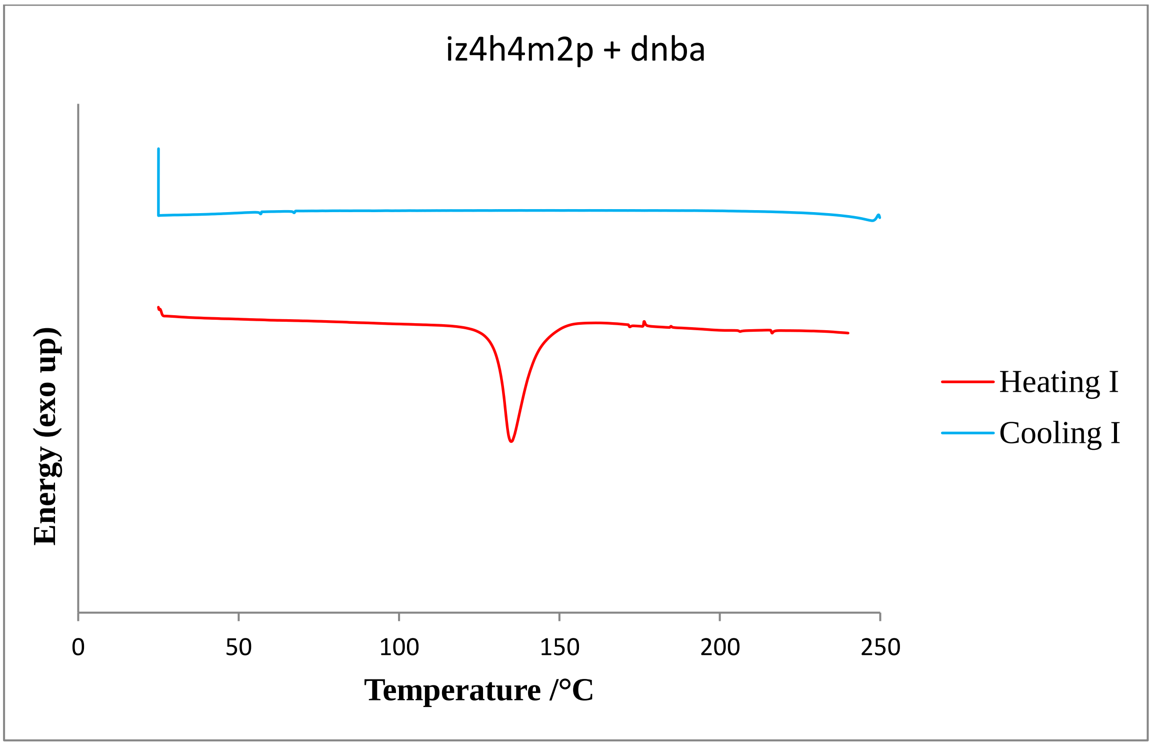

Figure S22 DSC curve for the cocrystal of **iz4h4m2p** + **dnba**.

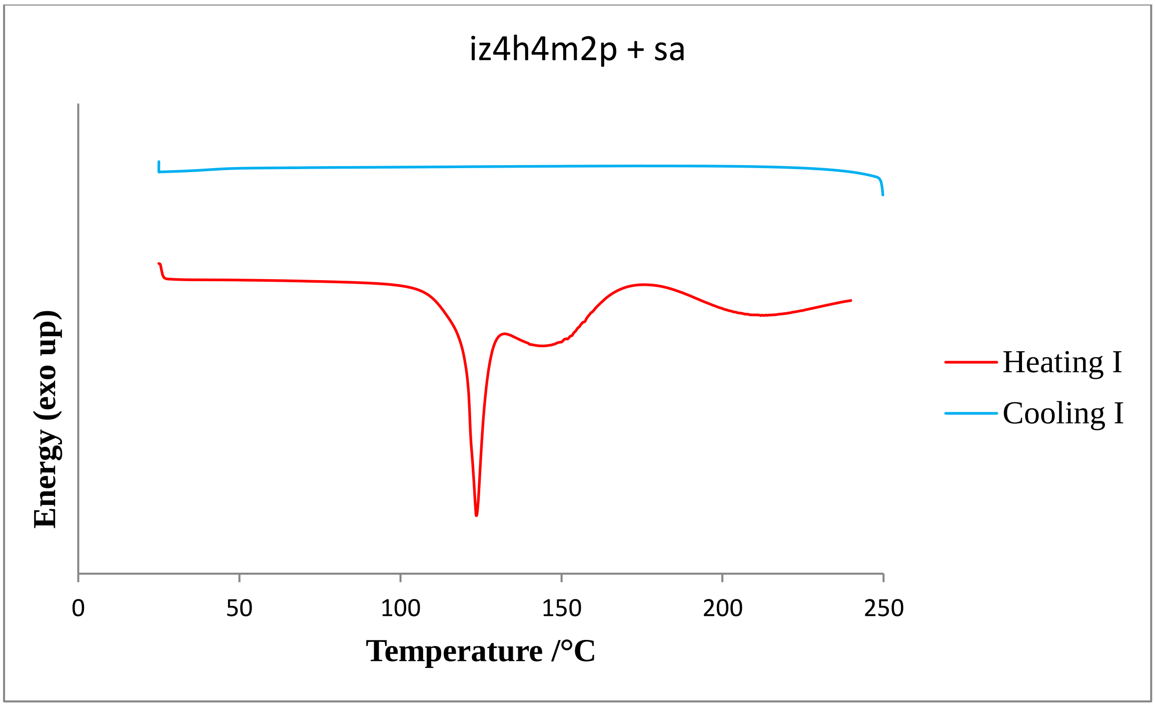

Figure S23 DSC curve for the cocrystal of **iz4h4m2p** + sa.

Table S2 List of cocrystals and moleculars salts of **inh** and Schiff-based derivatives of **inh** respectively

Refcodes of cocrystals and molecular salts of inh	Refcodes of cocrystals and molecular salts of Schiff-based inh derivatives
BAJXAP	CEFZEU
BAJXET	CETWEF
CAQPIX	EPISAY
ACUDAG	FADHOJ
ACUDEK	FADHUP
ACUDEK01	GIYMAF
ACUDIO	GIYMEJ
BICQAH	GIYMIN
BICQEL	GIYMOT
BICQUB	JILXIO
BICQUB01	JILXIO01
BIZMAZ	JILXOU
BOMBOW	JILYAH

BOMBUC	JILYEL
BOMCAJ	JILYIP
BUQTEP	JILYOV
BUVQER	LATKEY
COSKUT	LATKIC
DADLUS	LATKOI
EJOYEJ	LATLAV
FADGEY	LATLID
FADGIC	LEFQUK
FADGIC01	LIGXEG
FADGOI	NAKYOQ
FADGUO	NAKZIL
FADGUO01	OKAVED
FADGUO02	OKAVIH
FADHAV	OKAVON
FADHEZ	OKAVUT
FADHID	SAYNOX
FOSFIE	SAYNUD
FOSFOK	SAYPAL
FOSFUQ	SAYPEP
FOSFUQ01	SIKTEO
FOSMIL	UBILAW
FOSMIL01	UMUZAG
FOSMIL02	WOCFAX
FOSMIL03	XOPYEJ
GIYLUY	XOPYUZ
LATKUO	XOPZOU
LATLEZ	YUJJOE
LATSUW	YUJJOE01
LATTAD	YUJKAR
LATTAD01	YUJKEV
LODHIX	YUJNAU
LODHOD	YUJNEY
MOXNAR	YUJQUR

NAKZOR	YUJRAY
ORAWIO	ZECXEN
PEHFUF	ZUVWUK
PEHFUF01	ZUVXAR
PEZVAU	FEDRIT
PINJII	FEDTIV
PUMFOV	
PUMGAI	
PUMGOW	
QICJOE	
SETRIU	
SETROA	
SETRUG	
SETSAN01	
SETSAN02	
SETSAN03	
UDUJIP	
ULOSOG	
UQEKAF	
URUDER	
VAXROD	
VEGHOH	
VEGHUN	
VOPQEZ	
WETZAZ	
WIWNAU	
YEVBAF	
YIFXOB	
KEBLUC	
KEFXOM	
PEZVAU01	
